# Towards Internet QoS Provisioning Based on Generic Distributed QoS Adaptive Routing Engine

**DOI:** 10.1155/2014/694847

**Published:** 2014-09-17

**Authors:** Amira Y. Haikal, M. Badawy, Hesham A. Ali

**Affiliations:** Department of Computer Engineering & Control Systems, Mansoura University, Mansoura 35516, Egypt

## Abstract

Increasing efficiency and quality demands of modern Internet technologies drive today's network engineers to seek to provide quality of service (QoS). Internet QoS provisioning gives rise to several challenging issues. This paper introduces a generic distributed QoS adaptive routing engine (DQARE) architecture based on OSPFxQoS. The innovation of the proposed work in this paper is its undependability on the used QoS architectures and, moreover, splitting of the control strategy from data forwarding mechanisms, so we guarantee a set of absolute stable mechanisms on top of which Internet QoS can be built. DQARE architecture is furnished with three relevant traffic control schemes, namely, service differentiation, QoS routing, and traffic engineering. The main objective of this paper is to (i) provide a general configuration guideline for service differentiation, (ii) formalize the theoretical properties of different QoS routing algorithms and then introduce a QoS routing algorithm (QOPRA) based on dynamic programming technique, and (iii) propose QoS multipath forwarding (QMPF) model for paths diversity exploitation. NS2-based simulations proved the DQARE superiority in terms of delay, packet delivery ratio, throughput, and control overhead. Moreover, extensive simulations are used to compare the proposed QOPRA algorithm and QMPF model with their counterparts in the literature.

## 1. Introduction

Increasing steadily gaining popularity of mobile phones, VoIP, IPTV, cloud computing, as well as sensor networks that interoperate with Internet creates a large demand for QoS support for future Internet applications [1]. The main motivation behind the design of the next generation Internet is convergence, that is to say, making the Internet the common carrier for all kinds of services. The Internet is destined to become the ubiquitous global communication infrastructure [2].

In the beginning, the Internet used the public switched telephone network (PSTN) telecommunications (TelCo) infrastructure. The major interest was on “*where issue*” which means where packets should deliver. Now the TelCo industry has started to use the Internet infrastructure as a backbone, and with the advent of multimedia applications people became aware of the “*how will issue*” (or, “quality of service (QoS)”). Generally, QoS can be defined as the ability to create various traffic management mechanisms in the network to differentiate between different classes of services and to provide some level of assurance and performance optimization that can affect user perception [3]. QoS has become one of the most important issues in the next generation network (NGN) [4].

The Internet exists in order to transfer information from source nodes to destination nodes. Simultaneously, diversity of Internet services has become very competitive and end users are demanding very high quality services from their service providers. Accordingly, to accommodate service quality, Internet service providers (ISPs) have to provide interconnections more efficiently. Thus, one of the key issues in such a converged network is routing.

Routing is the process performed by routers to select the best path from the source node to a destination node in a network. The Internet traffic volume continues to grow at a massive rate; there may be a time when networks start to be congested on a regular basis. This situation has been the major force for innovation and development of different QoS routing solutions. Future Internet will embrace QoS routing as a basic functionality for QoS provisioning.

QoS-effective routing scheme can be efficiently designed to allocate resources in the network, allowing user constraints to be met and maximizing operator benefits, taking into consideration properties of the underlying network. In general, routing involves two entities, namely, the routing protocol and the routing algorithm. Although there has been historically close tie between both entities, it is beneficial to decouple them. The routing protocol has the task of dynamically identifying and communicating topological information [5]. Although proposals for a QoS routing protocol for Internet exist, still there is no Internet QoS routing protocol in the Internet. A critical basis for routing is routing computation algorithm that calculates the shortest path (SP) at each router for every known destination based on current topological information.

Open Shortest Path First (OSPF) protocol [6] is perhaps the most famous link-state routing extensively deployed throughout the last decade. OSPF provides best-effort Internet routing relying on a single arbitrary metric for path computation. OSPF does not guarantee optimal network utilization of available network resources due to a single path/single metric routing, which may cause partial congestion of the network.

The notion of QoS is a guarantee by the network to satisfy a set of predetermined service performance constraints for the user in terms of the end-to-end delay statistics, available bandwidth, probability of packet loss, jitter, and so on. QoS-based routing must extend the current routing paradigm in four dimensions. First, routers need information about available network resources. Second, we calculate optimal paths that fulfill a set of constraints. Third, opportunistic routing must shift traffic from one path to another as soon as a “better” path is found. Fourth, optimal path forwarding algorithms must support multipath routing [7].

QoS extensions to OSPF (OSPFxQoS or QOSPF) [8] provide comprehensive mechanisms to support QoS. But it poses the following limitations.

- OSPFxQoS used per-flow reservation via resource reservation protocol (RVSP) [9]. Resource reservation is not an appropriate method as overheads for setting up a reservation are simply too high.
- OSPFxQoS considered limited QoS constraints in the routing process. OSPFxQoS considered only bandwidth as a metric. It did not fully capture the complete range of potential QoS requirements.
- OSPFxQoS used precomputation routing algorithm that amortized the computational cost over multiple requests, but each computation instance is usually more expensive than in the on-demand case, as paths are computed to all destinations and for all possible bandwidth requests. Moreover, the accuracy of the selected paths may be lower.
- OSPFxQoS gets feasible path (not the optimal one) with minimum number of hops and supports requested bandwidth.
- The major shortcoming of OSPF and OSPFxQoS is lack of self-optimization. The self-adaptation mechanism is static. Routers disseminate information only when topology changes.
- OSPFxQoS is unable to readjust forwarding paths in order to lessen the impact of congestions or load-balance traffic to optimize the performance of the network.

Although the literature is plentiful of numerous QoS architecture, routing plays essential role in QoS provisioning. The main intention of this paper is to describe QoS-based routing issues and identify the basic requirements of QoS intradomain routing. This paper introduces a general formulation that combines framework and approaches for QoS provisioning based on the knowledge necessary of the service with minimal impact to routing infrastructure established upon OSPFxQoS routing engine architecture. This paper presents the following theoretical and practical contributions.

Theoretical contributions include the following.

- First, exploring through discussion that regardless of QoS architecture, performance optimization inside autonomous systems (AS) is an important building block in the QoS provisioning.
- Second, exploring that performance optimization can be achieved via proposing a generic distributed QoS adaptive routing engine (DQARE) architecture to overcome OSPFxQoS limitations. DQARE is a distributed software routing engine anticipated to exploit the full advantage of distributed hardware and improve scalability, overall performance, and resiliency. DQARE architecture is supplied with three unique features, namely, service differentiation, QoS routing, and traffic engineering (TE).
- Third, we address factors that affect perceived QoS, study Internet applications and recognize their QoS requirements, and organize applications into classes. Then, we introduce a general guideline for marking packets with a distinct code in order to differentiate between different types of services. Consequently, no resource reservation is required.

Practical contributions include the following.

- First, developing an efficient hybrid QoS path computation scheme which compared with state-of-art QoS routing schemes is unique in providing minimal complexity and low error decision rate. Our proposed routing scheme assumes that the true state of the network is available to every node. The computation scheme involves combining precomputation and on-demand multiconstrained routing algorithms to retrieve multiple paths for a QoS request.
- Second, we introduce a QoS multipath forwarding (QMPF) model. Multipath forwarding is a well-known approach to intradomain TE used to exploit path diversity provided by the proposed routing algorithms and to provide an autonomic congestion management mechanism. QoS provisioning while load balancing is still a challenge. From our knowledge, no researches had been devoted to such area. Several problems such as supporting per class delay guarantees, packet reordering delay, and protection among different service classes are yet to be addressed.

The rest of this paper is organized as follows.  Section 2 provides a general overview and discussion for QoS architectures.  Section 3 explores how to achieve Internet QoS provisioning regardless of QoS architectures.  Section 4 surveys related work.  Section 5 presents the proposed DQARE architecture. In Section 6, a general configuration guideline for service differentiation in conjunction with Internet application QoS requirements is presented.  Section 7 introduces the proposed routing framework and algorithms. The proposed QMPF model with algorithms is introduced in Section 8. In Section 9, we illustrate the applicability of the proposed solutions and present the experimental results.

## 2. QoS Architectures

There are three types of QoS, namely, perceived, assessed, and intrinsic QoS [10]. Perceived QoS (P-QoS) is a user-oriented QoS defined as the quality perceived by the users which depends on what the end points can do for the applications. It is mainly concerned with the software application industry not the network industry. Assessed QoS refers to the will of a user to keep on using a specific service. It is related to P-QoS and depends on marketing and commercial aspects.

Intrinsic QoS (I-QoS) is a network-oriented QoS concerned with what the networks can do for the applications. I-QoS may be described in terms of objective parameters such as delay, jitter, and loss. A key issue is how to provide efficient and fair routing so as to provide overall accepting service for most of the users. Most of QoS provision is offered in terms of I-QoS. It is mainly a technical problem dealt with by engineers, designers, and operators.

Supporting QoS in packet switching networks requires specialized infrastructure to be designed and developed that involves frameworks and new service models in addition to the existing best-effort service and resource management techniques that are based on the concept of dividing flows to traffic classes that are served with various QoS levels. This also requires traffic management schemes to be introduced, such as signaling, resource reservation, marking, packet classification, admission control, traffic conditioning, queuing, scheduling, and buffer management.

Two complementary basic approaches have been devised to guarantee QoS in data networks. The first is reservation based (state-oriented) approaches in which network resources are reserved in advance according to application's QoS request needs and subject to bandwidth management policy. The second is prioritization-based (stateless) approaches in which there is no resource reservation. Instead, traffic is classified and network elements give preferential treatment to classifications identified as having more demanding requirements.

Although the literature is plentiful of different QoS architecture, none of them become dominant or widely implemented on the Internet. There are two well-known proposed QoS architectures, namely, integrated service (IntSer) and differentiated service (DiffSer). For a more comprehensive discussion about different architecture, we refer the reader to [11].

In early 1990s, the IETF standardized the service types that build the first complete model of QoS assurance IntSer framework [12]. IntSer framework was developed based on certain key theoretical assumptions established upon reservation-based approaches. For flow awareness and achieving end-to-end real guarantees, IETF specialized a signaling protocol called RSVP [9]. RSVP was used by hosts to request specific QoS from the network without any limitation on the number of classes of service. IntSer established the foundation for QoS architecture and flow awareness; however, it suffered from complexity and scalability problems. IntSer cannot be ignored, regardless of these drawbacks; its features allow assuring QoS for each flow.

Afterwards, some researchers followed the direction of IntSer to solve its drawbacks and others favored to forego a different direction. Connectionless approach [13] decided to follow IntSer and mitigate the scalability problem via automatic detection of QoS requirement on the fly rather than using signaling protocol. It used traffic conditioners that consist of a classifier, admission control, and scheduler. However, automatic detection not always work probably which affects the robustness of the architecture.

The DiffSer architecture [14, 15] was an alternative QoS model developed by IETF to cope with the scalability problem faced by IntSer. DiffSer deployed prioritization-based approaches, abandoned flow level, and worked at class level, where a class is an aggregate of many flows. DiffSer aims to guarantee QoS per traffic class. DiffSer architecture used resource allocation quite different from IntSer. DiffSer approach used six bits in the Type of Service (TOS) field in the IP packet header, which has been transformed into DiffSer field (or code point) to encode the forwarding treatment (technically called Forwarding Equivalence Class). DiffSer was contemplated using limited classes of services (CoS) that makes sense to add new complexity in small increments to the existing best-effort service. A router can offer to packets two aspects of preferential treatment which are expedited forwarding (EF) and assured forwarding (AF) in addition to unclassified service (i.e., best effort). DiffSer does not require the soft-state concept and thus avoids session-level scalability issue faced with RSVP.

Although DiffSer solved the scalability problem of IntSer, it failed to provide end-to-end QoS provisioning [16], and there is no performance guarantee. In reality, the DiffSer has been deployed by some ISPs. Li and Mao proposed a novel flow-based scheme [17] established upon DiffSer. Such scheme ensured a constant proportion between the P-QoS by flows in different classes, regardless of the current class loads. The scheme is furnished with an estimator for the number of active flows, a dynamic weighted fair queuing (WFQ) scheduler, and a queue management mechanism. Nevertheless, this architecture still has a limited number of CoSs and complex operation.

Even if QoS is the subject of a great number of tutorials, papers, patents, recommendations, and standards, there is a question which still needs an answer: “does procuring an oversupply bandwidth (overprovisioning) will solve all QoS challenges?” Xiao [3] and Montanez [18] stated that (i) there is no need for QoS mechanisms and all you need to achieve QoS is sufficient capacity; (ii) overprovisioning works satisfactorily and the service differentiation will not be able to create perceivable differentiation either in normal or abnormal conditions; (iii) best effort can provide good enough performance for most applications in the developed countries; (iv) it is commercially difficult to install QoS in a network.

However, there are raised objections: (i) bandwidth guarantee is indeed a key component for offering QoS. But purchasing an oversupply of bandwidth will not solve all service-quality challenges; bandwidth optimization and possible future trends and requirements of new services must be considered; (ii) if utilization is observed to be higher than the acceptable threshold for a particular link type, so it is easy to trivially add bandwidth/capacity to the network. However, in large networks, where the utilization can be further impacted by routing, adding bandwidth is much more complex than this simple single-link network, and (iii) lack of differentiation among services leads to difficulty to sell QoS which is the fundamental cause of the commercial challenges.

Actually, an important lesson is learned from the above discussion. DiffSer traffic management is not the primary way to enable QoS, introducing DiffSer traffic management mechanisms alone cannot provide a satisfactory QoS solution, and too much complexity may have crept into the network. DiffSer traffic management is good for creating some service differentiation when there is congestion, but how often real-world networks are like that is unverified. In a more insistently QoS environments, service differentiation, QoS routing, and optimization using traffic engineering (TE) techniques have become the only viable alternative.

## 3. Proposed QoS Provisioning Methodology

The Internet today is going through new generations of innovative and fast applications that need high performance demands on the Internet infrastructure. Wójcik and Jajszczyk [11] stated that progress in access network capacities is far greater than in core networks and the bandwidth is always consumed. There may be a time when networks start to be congested on a regular basis and efficient and feasible QoS provisioning methodology might then be needed. To keep pace with the continuous demands, not only does the bandwidth need to be increased, but also the routers that power the Internet have to evolve architecturally and be furnished by congestion management schemes.

**Figure 1:**
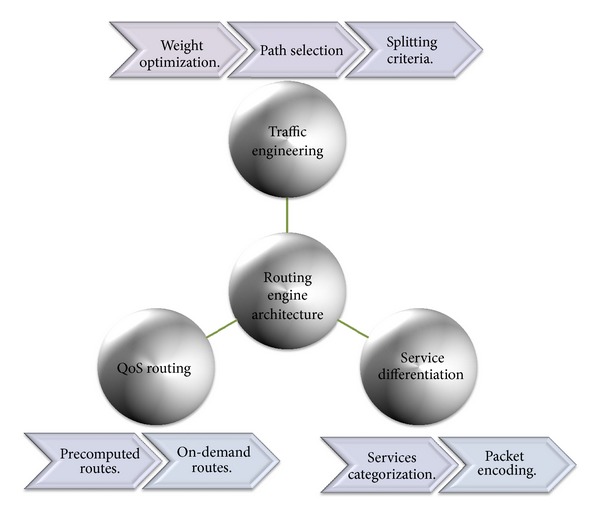
Internet QoS provisioning methodology.

“How QoS is possible in Internet, and how can it be achieved in an efficient and reliable manner?”. At the core of the answer, in a realistic sense, and to provide acceptable QoS performance, performance optimization inside autonomous systems is an important building block in the deployment of QoS. The majority of QoS publications focused on QoS architecture and traffic management schemes, although it only affects the performance of specific link of routers. The work presented in this paper takes a different approach. More effective Internet QoS provisioning can be achieved, as shown in Figure 1, via introducing a generic QoS routing engine architecture furnished with three intertwined traffic control schemes blocks that contribute to QoS provisioning.

The three-dimensional traffic control schemes contain service differentiation, unicast intradomain QoS routing algorithm, and traffic engineering (TE). These schemes must work in tandem for providing efficient services. These traffic control schemes can be used to reduce or prevent congestion and introduce a bigger impact on QoS than traffic management schemes as they affect traffic performance network wide. The coordination among these traffic control schemes is significant and utilizing service differentiation will assist in better perceived QoS.

*Service Differentiation.* ISPs are deploying more resources to handle the emerging applications. In order to lessen the amount of deployed network infrastructure and resources, differentiation of ISP offering services is needed. Service differentiation via setting the IP header TOS field must be designated in order to give better service when it is available. It is important to define a wide range of services, each with its own requirements. Accordingly, service differentiation has to enable deployment of scalable service discrimination in the Internet without the need for per-flow state and signaling protocols.

*QoS Routing Algorithm.* The problem of QoS routing has been the center of attention in both academic and industrial communities for some time. There is a need for additional capabilities in the IP routing world and performance management tools to determine optimal paths that satisfy a set of constraints. For QoS provisioning, the way of path selection should also be QoS-aware which means identifying an optimal route that meets multiple constraints which is more complex than best-effort routing. 

*Traffic Engineering (TE).* Enhancing the performance of QoS routing at both traffic and resource levels is the major objectives of QoS intradomain TE. TE is thoughtful as an aspect of Internet network engineers. TE is the process of optimizing the operational performance of networks flows through better control, at both network flows and resource levels. One of the major intradomain TE techniques is multipath forwarding (MPF) using traffic splitting that is routing traffic in a way that can effectively maximize utilization of network resources, supplying path protection, reducing blocking capabilities, minimizing delays, and increasing throughput. Thus, TE regarding intradomain routing can be defined as supplementary to the routing infrastructure and QoS provisioning.

## 4. Related Works

QoS-based routing has been recognized as a missing piece in the evolution of QoS-based service offerings in the Internet. Special attention must be given to new powerful architectures for routers in order to fulfill the demanding critical role in QoS provisioning. This section covers a literature review and opens challenges for router architectures, service differentiation, QoS routing algorithms, and multipath forwarding mechanisms.

### 4.1. Router Architecture

The basic key functionalities in an IP router can be categorized into three functions (or planes): (i) route processing (how you direct bits), (ii) packet forwarding (how you move bits), and (iii) management services which includes management applications, protocol policies, and QoS. Router architectures have experienced three generations in terms of hardware and software [19, 20].

The first generation of IP router was built around conventional computer architecture [21]. Unfortunately, this simple architecture produced low performance as the three planes competing for the same processing unit.

In the second generation IP routers, improvement was introduced to increase the system throughput by distributing the packet forwarding operations by using multiple processors with on-demand lookup route caching. However, the frequent changes in network topology in the core of the Internet caused the cache entries to be invalidated frequently, resulting in smaller hits [22]. The third generation of routers introduced a hardware-forwarding engine and replaced shared bus by a high-speed crossbar switch with the aim to achieve higher throughput [23]. This architecture is still limited by the drawbacks of cache schemes.

Actually, structure of previously routers' architecture has an architectural limitation when it comes to meeting future requirements. The network performance degrades as the volume of traffic increases. These architectures relied on shared resource for all access and transfers and there is a centralized arbiter or scheduler responsible for granting access to the resource. The modularity of the hardware and the software is a key to the implementation of a modern router.

For QoS provisioning, next generation routers (NGRs) should be dependent upon fully distributed architectures in which partitioning the functions physically, logically or even both as clearly as possible to simplify system design and testing and achieve throughput and robustness as well as system availability. Distributed processing architecture is a combination of all the techniques discussed above.

A distributed routing engine comprises the modules running on different cards of a router. The main objective is to overcome the previous limitations of processing, memory bandwidth, and bus bandwidth via distributing overall processing and buffering capacity over the CPU and network interfaces equipped with processing power and buffer space. The functions of the forwarding engines are integrated into the interface cards in the distributed mode. Processing load gets distributed, ensuring faster and more reliable communication. 

*Open Challenges.* NGRs are fitting QoS requirements that lie in the critical path of data flow. The software architecture for next generation routers should therefore be much more distributed in order to be scalable and to take full advantage of the distributed hardware platform entailed by the switch fabric.

### 4.2. Service Differentiation

Internet architecture is characterized by fairness which means that all kinds of applications are fairly shared network resources. Therefore, there is only one forwarding treatment deployed that cannot bear QoS oriented applications. QoS deployment needs a methodology to classify and differentiate between different applications. The Internet layer of TCP/IP stack on which routers operate must be able to distinguish between different classes of services.

Routers are able to distinguish between packets. The IP protocol provides a facility for upper layer protocols to convey hints to the Internet Layer about forwarding path behaviors. This facility is first addressed by the TOS field in IP header [24].

DiffSer standards [25] replaces the IP TOS field by 6-bits code points (also called differentiated services code point (DSCP)) and two bits currently unused to indicate the forwarding equivalence class (FEC). The DSCP identifies a specific traffic class and implies that all the packets identified with the same DSCP should receive the same treatment. Considerable debate took place on the allocation of these 6-bits code points. Following RFC2474 [15] and RFC4594 [25] general guidelines, the DSCP field can convey 64 distinct code points divided into three pools: 32 DSCPs are dedicated to standard recommended code points (Pool 1), 16 to be reserved for experimental and local use (Pool 2), and the other 16 (Pool 3) to be initially available for experimental and local use but may devote again to standard actions if Pool 1 is ever exhausted. 

*Open Challenges.* DiffSer provided limited set of traffic classes and therefore a risk is the aggregation of nonhomogeneous traffic. Class based classification with a limited number of classes does not guarantee that flows classified with a higher priority will really observe a better quality of service than lower priority ones, due to the fact that the distribution of active flows in individual classes might be different. There is a crucial need for scalable service differentiation guidelines.

### 4.3. QoS Routing Algorithms

QoS-aware routing algorithms aim to find a path that obeys multiple constraints. Generally, traditional routing paradigm can be extended to support QoS by considering two important issues: first, choosing and distributing relevant QoS measures and, second, how to compute routes based on the information collected. Metric selection is very important in the sense that “the metrics must represent the basic network properties of interest.”

In QoS arena, routing metrics can be broadly divided into two classes. The first class is static (cumulative) metrics which value does not change over time. These metrics can be classified into (i)* additive parameters* (e.g., delay, hop count, and jitter), where the cost of a path is the sum of the individual link values along that path and (ii)* multiplicative parameters* (e.g., packet loss) which can be approximately transformed into additive by taking the logarithm of the multiplicative measures on each link. The second class of routing metrics is dynamic metrics (also referred to as a bottleneck or concave or min/max metrics), where metric's value changes over time with each request (e.g., bandwidth). Many researches considered only two additive QoS constraints [26].

The constraints associated with dynamic parameters can be handled by* postprocessing* which finds multiple paths from source to destination that satisfy set of static constraints and then select one path from these paths such that all the other dynamic parameters are satisfied [27]. Otherwise, preprocessing can be used for pruning from the graph all the links that do not satisfy constraints and then search for a feasible path [28]. In practice, the constraints on additive QoS metrics are more challenging, and, therefore, without loss of generality, the QoS metrics are assumed to be additive [29].

QoS multiconstrained path selection with additive parameters is an NP-complete problem that cannot be exactly solved in polynomial time [30]. However, Kuipers and Mieghem showed that the “worst-case” behavior is very unlikely to occur in practice and thus exact QoS routing algorithms seem feasible [31].

QoS-aware routing algorithms in the literature can be classified according to the path computation triggering criteria into precomputation QoS routing algorithms (PQRA) and on-demand computation QoS routing algorithm (OQRA). A comparison between the two paradigms is conducted in Table 1.

Most QoS routing algorithms presented in the literature used OQRA. OQRA may cause an insufferable computational overload in the high-speed next generation networks. OQRA needs to be called each time a new demand needs to be routed.

OQRA can be classified into four classes. The first class is heuristic algorithms [26, 32–35], where QoS routing is NP-complete; it demands heuristics in global optimization that help decide which one of a set of possible solutions is to be examined next. The second class is *ε*-approximation algorithms [36–38] which give good approximate solutions to the problem which may not necessarily be exact. The third class is the exact algorithms [39, 40], where exactness can be reached by computing all possible paths between source and destination where the exact path is guaranteed to be found. The last class is metaheuristics which used metaheuristics, such as ant colonies [41] and genetic algorithms [42].

TAMCRA [33] is a heuristic algorithm that is based on three concepts: (i) a nonlinear measure of the path length, (ii) a *k*-shortest path approach, and (ii) the principle of nondominated paths. TAMCRA aims only at finding a feasible path (not optimal). The major drawback is that if an intermediate node found at sub-path better than the stored *k* paths, it replaces stored paths even it has much longer post-path to destination. In other words, stored prepaths may misguide the search towards the shortest path. TAMCRA has a worst-case complexity of *O*(*kN*log⁡⁡(*kN*) + *k* ^3^ *mM*) per request.

To overcome the TAMCRA drawbacks, Korkmaz and Krunz [34] presented a heuristic algorithm called HMCOP, which tried to find an optimal path within the constraints by using the nonlinear path length function for feasibility. HMCOP searched for the path that not only is feasible but also minimizes the value of a primary QoS attribute. HMCOP executed two modified versions of Dijkstra's algorithm in the backward and forward directions. In the backward direction, HMCOP computed an estimate of how suitable the remaining subpaths are. In the forward direction, HMCOP used a modified version of Dijkstra's algorithm. This version heuristically determined complete path by concatenating the postpath from source to intermediate nodes and the estimated prepaths. HMCOP provided a preference rule for choosing paths. The drawback of HMCOP is that postpath may misguide the selection of prepaths. HMCOP had the worst-case complexity of *O*(*N*log⁡⁡*N* + *mM*).

SAMCRA [39] is the exact successor of TAMCRA to obtain multiconstraint optimal path. SAMCRA was based on four fundamental concepts: a nonlinear measure of the path length, *k*-shortest path approach with attainable bound for *k*_max⁡_, the principle of nondominated paths, and the concept of lookahead to calculate attainable lower bounds and reducing search efforts. SAMCRA is considered an effective algorithm; however, the major drawback of SAMCRA resides in its complexity which is *O*(*kN*log⁡⁡(*kN*) + *k* ^2^ *mM*).

Retrieving multiple paths subject to multiple constraints was addressed in [27]. The algorithm called A* Prune finds not only one but also multiple shortest paths satisfying the constraints listed in order of increasing length. A* Prune algorithm may be considered similar to the SAMCRA algorithm [39] except that it relied on linear path length. The worst-case complexity of A* Prune is *O*(*N*!(*m* + *h* + *N*log⁡⁡*N*)), where *h* is the number of hops of the retrieved path.

To compute feasible paths, Bellabas et al. [35] proposed two fast heuristic algorithms with less combinatorial complexity. The first heuristic algorithm is the hop count approach (HCA) that computes paths with the smallest hop count. The second is called metric linearization approach (MLA) that used a combination of QoS metrics. To store multiple paths at each intermediate node, they proposed a modification to Yen's algorithm [43] which was generalized by Lawler in [44]. HCA and MLA stop at the first feasible path they find thus reducing their execution time. However, they cannot obtain optimal paths.

Shin et al. proposed MPLMR [26] which is a heuristic multiconstraint QoS routing scheme. MPLMR used the same concepts as TAMCRA and HMCOP to store a limited number of subpaths between the source node and each intermediate node. MPLMR used an improved “lookahead” method to estimate full path length. Sanguankotchakorn et al. [45] proposed an algorithm (RMCOP) to find feasible path that somewhat satisfies multiple constraints. They proposed a relaxed lookahead algorithm. However, the computational complexity of the proposed algorithm is large.

Contrarily, precomputation schemes use an offline procedure. The overall computational load of such scheme is reduced, especially when the rate of QoS request arrivals is much higher than that of significant change in the network state. However, temporal conditions like congestion in the network and routing between two subsequent updates makes the routing decision be calculated based on inaccurate information resulting in nonoptimal path selection.

QoS routing mechanisms and OSPF extensions [8] focused on the algorithms used to compute QoS routes and the necessary modification to OSPF to support QoS. OSPFxQoS deployed a precomputing routing algorithm that amortized the computational cost over multiple requests with the motivation to get a feasible path with minimum number of hops (low resources) and support requested bandwidth for all possible QoS requests. The presented algorithm has a computational complexity compared with Bellman-Ford algorithm [46] but with limited QoS constraints consideration in the routing process.

An approach for pre-computation of multi-constrained path (PMCP) was proposed in [47]. It computed a number of QoS coefficients based on which linear QoS function was computed and then constructed different shortest path trees to compose the routing table. PMCP proposed algorithm has a complexity of *O*(*B*(*m* + *n*log⁡*n* + *n*)). Jin [48] proposed precomputation algorithm called limited selective flooding (LFS) routing algorithm. LFS considered an MCP problem with imprecise additive link state information. Authors in [49] presented *O*(*Km* + *n*log⁡⁡*n*) time *K*-approximation precomputation algorithm. Afterwards, in [50], the authors reduced the computational complexity. 

*Open Challenges.* NP-completeness of the QoS routing problem leads to only few exact algorithms proposed in the literature. The difficulty of QoS algorithm lies in its computational complexity and success rate. Designing QoS routing algorithms with low complexity, high performance, and high success rate is still an open issue.

### 4.4. Multipath Forwarding (MPF)

Two major issues had drawn attention in recent years regarding TE. The first issue is to provide a TE mechanism to effectively develop a routing optimization that enhances network service capability without causing network congestion. The second issue is achieving resiliency via introducing a TE solution that minimizes the impact of nodes and link failure [51].

The first and foremost question of providing congestion control and QoS degradation mechanisms is “how to explicitly control traffic distribution inside an AS?”. At the core of the answer lies intradomain TE. Extensive deployment of intradomain routing protocols such as OSPF has drawn an ever increasing attention to Internet TE in recent years.

The major TE technique is multipath forwarding (MPF), also called multipath routing, using traffic splitting and path protection. Multipath intradomain forwarding can be used to handle traffic congestion inside a domain. Routers must be equipped with multipath forwarding mechanism to perform traffic forwarding. So, multipath load distribution is engineered by two key capabilities: (i) control-plane extension: deploying a routing algorithm to compute multiple paths and (ii) data-plane extension: providing a load distribution model to the forwarding process engineered by two key functionalities, namely, traffic splitting and path selection.

**Figure 2:**
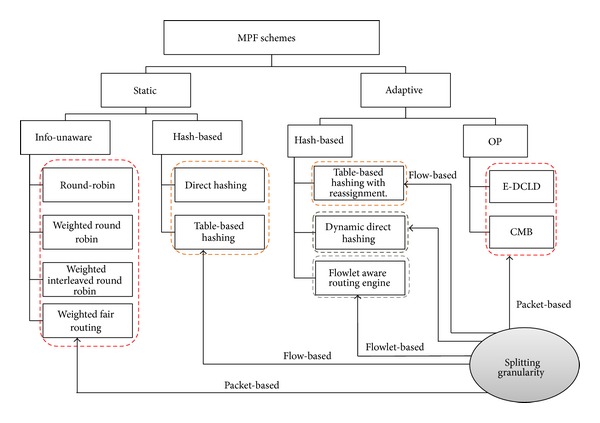
LDM classification.

Although, Internet topology involves path diversity, it is underexploited, as 30–80 percent of the time an alternate path with lower loss or a smaller delay exists and is never exploited [52]. Fortz et al. reported that load balancing improves the network service capability by 50%–110% compared to single path routing [53–55]. MPF had attracted a large body of literature. A number of load distribution models had been proposed and studied. LDMs in the literature can be coarsely categorized, as shown in Figure 2, into the following two groups: static load distribution model (SLDM) and adaptive load distribution model (ALDM).

Information less models use packet as the traffic unit. It makes a raw decision on distributing traffic without taking into account packet information [56–58]. Static hash-based models choose the path in terms of flows instead of packet-per-packet splitting. Such models process a flow as an allocation unit that must be traversing the same path. Such models calculate a hash over selected fields in the packet header. There are a variety of hash-based models such as direct hashing (DH), table-based hashing (TH) [59, 60], and fast switching (FS) [61].

ALDMs take into considerations network conditions such as average path delay, link utilization, packet interarrival time, and capacity in path selection. Chim and Yeung [62] proposed adaptive hash-based LDM named table-based hashing with reassignment (THR) that helps to redistribute the traffic load. THR improves the TH algorithm by combining actual load sharing statistics and dynamically reassigns some active flows (bin-to-path mapping) from the overutilized paths to underutilized paths. However, out-of-order packets delivery is still present.

Kandula et al. [63] proposed flowlet aware routing engine (FLARE) that operated on flowlet. In order delivery of flows can be achieved via assigning flows to any available path, if the time between two successive TCP packets is larger than the maximum delay difference between the parallel paths. However, the common limitation lies in the estimation process which may be inaccurate at high packet arrival rate and this may yield load imbalance.

Tian et al. [64] provided network-wide load balancing performance by introducing link-criticality-based ECMP routing (LCER) algorithm. LCER selects path based on the link's average expected load, link capacity, and the path's length. LCER provides in-order packet delivery and the lowest average end-to-end packet delays.

Y. Wang and Z. Wang [65] considered multipath routing as an optimization problem (OP) with an objective function that minimizes the congestion of the most utilized link in the network. However, they did not consider the quality of the selected paths. Banner and Orda [66] proved through comprehensive simulations that multipath solutions obtained by optimal congestion reduction schemes are fundamentally more efficient than solutions obtained by heuristics. They formulated MPR problem as an optimization problem of minimizing network congestion. They established a polynomial time algorithm that approximates the optimal solution by a (small) constant approximation factor. However, this method is not a direct solution of potential of QoS necessities.

MPF can provide a unique solution to congestion problems by utilizing the available resources in an adaptive way to the dynamics of traffic demands. One way to prevent congestion is to control the delay within the network. Thus, network capacity and QoS provisioning need a new load distribution model aiming to minimize the difference among path delays, thereby reducing packet delay, jitter, and risk of packet reordering without additional network overhead.

Motivated by the scarceness of solutions to efficiently control packet delay for real-time traffic, Prabhavat et al. developed enhanced delay controlled load distribution model (EDCLD) [67]. EDCLD is an interesting packet-based delay-controlled LDM developed to strike the lower delay and packet ordering to utilize parallel paths for multimedia data transmission and real time applications. Prabhavat et al. formulated a delay-aimed problem model to figure the optimal load ratio and its corresponding path. EDCLD used iterative method to calculate optimal traffic-splitting vector so that maximum path delay can be minimized. The trick of EDCLD is to reduce the difference between path delays by using adaptive load adaptation algorithms that gradually, according to the number of paths, approach traffic-splitting vector among the paths. EDCLD decreased load assigned to the path with the largest delay and increased load by the same amount to the other path with the smallest delay. In the path selector, they implemented the SRR load sharing algorithm [68].

Li et al. [69] proved that the optimization problem of EDLCD is convex. They proposed a convex based method (CBM) that defines the optimal load ratio in one shot rather than gradually approaching algorithm used by EDLCD. Their proposed scheme outperforms EDCLD specifically with instability and large number of paths. Their proposed scheme also relied on the SRR load sharing algorithm.

Packet-based scheduling can achieve very accurate splitting percentages and adds very little extra overhead. However, it suffers from major problems which are packet reordering and TCP throughput degradation.

The major drawback of static hashing is a load imbalance problem due to an inability to deal with variation of the flow size distribution. Thus, one major challenge of TE is supplementing adaptive control capabilities that adapt quickly to significant changes in a network's state.

Majority of ALDMs is linked to TCP-traffic only and focus on load balancing efficiency and packet order preservation. These schemes are unsuitable for QoS oriented applications as they cannot guarantee low delay and packet ordering. In addition, Martin et al. [70] studied multistage network architecture. They discover that all pure hash-based algorithms have one serious problem, namely, traffic polarization effect (TPE). Also, Shi et al. [71] proves that pure hash-based algorithms cannot well balance load in the face of the highly skewed flow-size distributions in the Internet.

On the other hand, EDCLD and CBM are considered effective for real-time applications MPF. However, EDCLD used a gradually approaching method that needs several rounds, depending on the number of paths, to reach convergence and cannot handle paths instability. CBM limitation lies in solving a nonlinear optimization problem which can incur a significant computational overhead when performed on a per-packet basis. 

*Open Challenges.* There is a crucial need for an effective MPF scheme that optimizes the network, with the joint goals of avoiding network congestion and ensuring QoS provisioning.

## 5. Proposed DQARE Architecture

The proposed DQARE architecture is based on extending the current Internet routing model of the OSPFxQoS routing engine to support strict QoS. DQARE architecture inherits the cons of the OSPFxQoS routing engine architecture explored in [8, 72] and nullifies the drawbacks of OSPFxQoS architectures. It is worthy to divide the control plane functionality among modules on different cards of router, namely, control card and line cards, exploiting the power of next generation routers to move some control functions to line cards.

**Figure 3:**
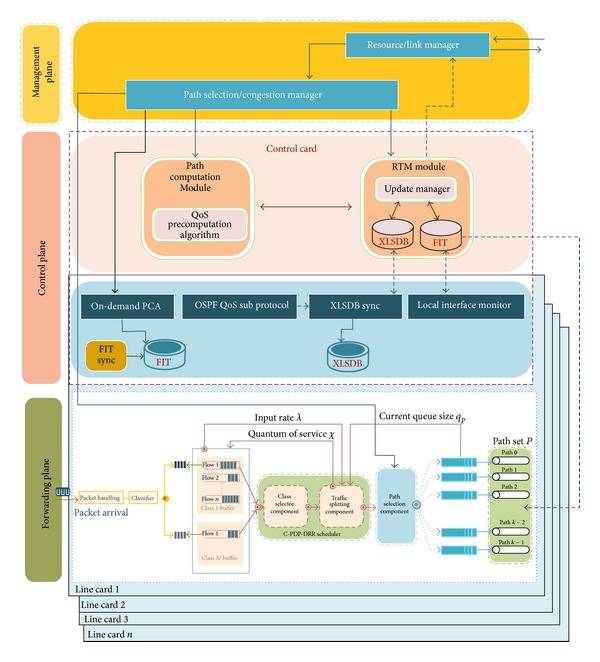
Proposed DQARE architecture.

Basically, as Figure 3 depicts, the proposed software architecture of a router is composed of three planes connected by interfaces: (i) forwarding plane (or data plane): the main task of this plane is to forward flows in a way that prevents congestion. So, forwarding plane is supplied with MPF model. (ii) Control plane hosts routing protocols that are responsible for establishing routes within an AS, routing table management, sending and receiving link state updates and computing shortest paths. The control plane computes forwarding information table (FIT) from one or several routing tables that are used by forwarding plane. Some of the functions of control plane are implemented on the control card and others on the line cards. (iii) Management plane handles network management applications, protocol policies, and QoS. This plane performs congestion and path management.

For QoS provisioning, the most significant parts in adoption are relying on prioritization approach instead of reserving resources as in OSPFxQoS, the changes to the routing algorithm in the control plane that computes diverse paths to the forwarding plane subject to two additive constraints, the implementation of load balancing, and protection algorithms to the forwarding plane.

**Figure 4:**
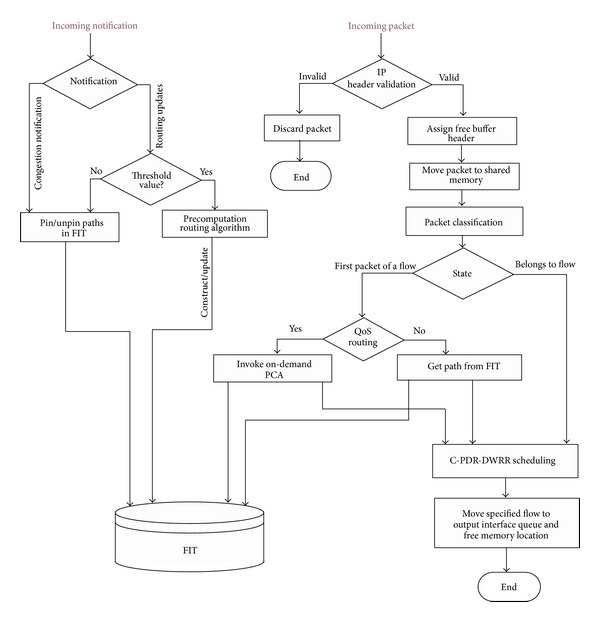
DQARE architecture functional flow.

The functional flow of the proposed framework is illustrated in the flowchart depicted in Figure 4. The functional blocks of the proposed architecture are as follows.

*Packet Handling.* Packet handling involves the following functions:* IP Packet Validation*: as a packet enters an ingress port, the forwarding logic verifies all layer 3 information (header length, packet length, protocol version, checksum, etc.) and* Route Lookup and Header Processing:* the router then performs on-demand path computation using the packet's destination address and QoS constraints to determine the output of the egress port(s) and performs all IP forwarding operations (packet lifetime control, header checksum, etc.).

*Classifier.* QoS applications operate on packet flows so the routers must be able to classify individual traversing packets. Actually, it is very difficult to guarantee the delay bound to specific flows without flow isolation. DQARE architecture relies on marking packets using DSCP bits to identify the class of traffic. Flows are firstly grouped in traffic classes by the classifier. Classification of traffic provides more equitable management and more stability in the use of pure priorities. We consider the objective of service differentiation to ensure low packet delay for streaming applications. All packets in the same class are treated equally by the* C-PDR-DWWR* scheduler.

*Path Computation Module.* Assuming the router maintains link state information of the entire domain, DQARE architecture uses two types of routing algorithms, firstly PQRA implemented in the control card. PQRA precomputes the routing paths from a node to all destinations subject to two additive metrics, prior to receiving the requests, and stores the QoS information in its routing table. When receiving a connection request with QoS requirements, OQRA implemented on line cards computes a path from offline precomputed routing table and finds an optimal or a feasible path for this request if found. On-demand path computing can be running again in the congestion state with benefit of ensuring a strict bound on the computational load.

*QoS Load Balancing Module.* multipath Intra-domain forwarding is used to handle traffic congestion inside a domain. DQARE architecture is equipped with MPF mechanism to perform traffic forwarding. The load balancing module consists of combined proportional delay prioritization and dynamic weighted round robin (C-PDR-DWWR scheduler) and path selection component.

*C-PDP-DWRR* is work-conserving scheduler that works in two phases. In the first phase, class based priority scheduling is performed to select class to serve from multiple classes. Also, the first phase achieves proportional queuing delay differentiation protection among various classes. Proportional service differentiation, originally proposed by Dovrolis et al. [73], is perhaps the best known effort to enhance class-based services with relative guarantees.

Dovrolis et al. proposed proportional delay differentiation (PDD). The network traffic is grouped into *N* classes of service which are ordered, such that class *i* is better (or at least no worse) than class *i* − 1 for 1 < *i* ≤ *N*, in terms of queuing delays. *d*_*i*_, *δ*_*i*_ denote the average queuing delay and delay differentiation parameter (DDP) value. The PDD model aims to control the ratios of the average class queuing delays based on the delay differentiation parameters (DDPs) {*δ*_*i*_ : *i* = 1,2,…, *N*}. The PDD model requires that the ratio of average delays between two classes *i* and *j* is fixed to the ratio of the corresponding DDPs as follows:

(1) $$\begin{array}{c} \frac{d_{i}}{d_{j}}=\frac{\delta_{i}}{\delta_{j}}. \end{array}$$

Higher classes provide better service, that is, lower queuing delays, and so *δ*_1_ > *δ*_2_ > ⋯>*δ*_*N*_ > 0.

In the second phase, the traffic splitting component defines the amount of traffic (*χ*) forwarded on the selected path. Within the selected priority class, flows are scheduled in DWRR manner [74]. DWRR, on the other hand, allows higher priority queues to send a predetermined amount of data during a service round. Each queue is configured with a quantum of service (*χ*) and a deficit counter (DC). The scheduler also has the task of path delay adaptation by decreasing quantum of service (*χ*) on the path having the largest estimated end-to-end delay and then increases the quantum of service (*χ*) on the path having the smallest estimated end-to-end delay by the same amount of the reduced load.

*Extended Link State Database (xLSDB).* It consists of link state information. This information includes both static (delay, jitter, and loss rate) and dynamic metrics (current available bandwidth) of the whole topology. 

*Path/Congestion Manager.* It selects a path for a request with particular QoS requirements and manages it once selected; that is, it reacts to link or reservation failures. It finds alternative paths by invoking OQRA again in the case that the used path becomes unavailable. It invokes the traffic splitting component to select a flow to be shifted during congestion. This module also manages congestion handling actions when a congestion notification delivered from designated router (DR) or any link becomes congested and its associated queues reach their threshold level. 

*Routing Table Manager (RTM) Module.* The main task of the RTM is to build FIT that stores, if existing, multiple routes to the same destination from precomputation routing algorithms. It contains update manager module which determines when to advertise local link state updates and when to perform QoS path precomputation and control the paths within routing table; that is, it activates paths that meet the requirements and deactivate routes that currently does not meet the requirements. It contains xLSDB that contains information about the current state of the network. 

*Resource/Link Manager.* Its main role is to manage individual and bundled interfaces. It monitors the load on resources, handles the up/down status, and receive notifications from designated routers. 

*Local Interface Monitor* in the control plane handles the up/down status of each router interface.

**Figure 5:**
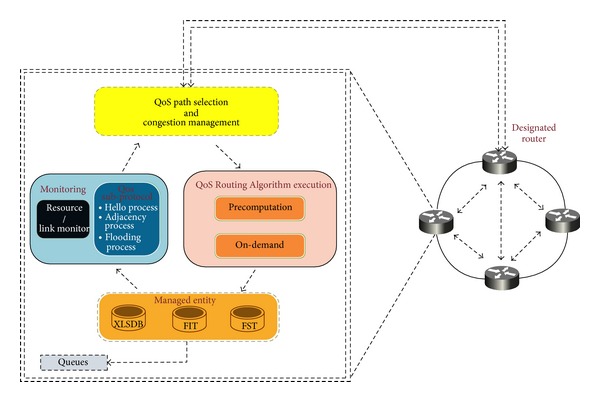
Self-adaptation mechanism.

As shown in Figure 5, DQARE architecture exploits GANA [72] self-adaptation mechanism which is a typical example of a protocol-intrinsic control loop. The OSPF protocol acts as a virtual distributed decision element (DE) scattered all over the domain. GANA provides different types of basic network services such as autonomic routing and advanced services such as QoS management.

DQARE architecture provides the self-adaptive control loop to prevent instantaneous congestion, which involves invalidating congested routes in the FIT, using other available paths if they exist, precomputation of routes whenever a threshold of updates is reached, a global resynchronization of xLSDB, and recalculations of the routing tables. This scheme seems to make a comprehensive overhead; however, global, reactive response makes the convergence of the control loop acceptable because each flooding message is sent only once instead of by all interfaces, so it reduces the traffic in the network and the proposed framework enables direct communication among line cards belonging to the same OSPF area which makes improvement over traditional architectures which yields that the reaction to failures is not a lengthy process.

The autonomic management components involved in the control loop are (i)* QoS path selection and congestion management:* responsible for sending and receiving notifications to/from the designated router (DR), (ii)* monitoring entity:* monitoring the local interfaces, links, and notifications. It can be implemented via the Hello protocol, (iii)* QoS routing tables execution:* implemented via precomputation algorithm that can be executed periodically or after receiving n-updates, and (iv)* managed entities* which control loop effects. It consists of FIT and extended LSDB (xLSDB) with a predefined set of actions to be performed such as sanction and neutralizing routes in FIT.

## 6. Service Differentiation Guidelines

QoS provisioning for various applications requires: (i) studying Internet applications and recognizing their QoS requirements, (ii) organize applications into classes and (iii) differentiate between different types of services by marking packets with a distinct code so that they receive certain kinds of treatment from routers.

### 6.1. Internet Traffic Classification

Traffic flowing in a network can be divided into two groups, network-oriented traffic and user-oriented traffic [25]. The network-oriented traffic group is divided into three service classes, namely, network control function, OAM (operations, administration, and management) for network configuration, and management functions and signaling to control applications or user endpoints. User-oriented traffic can be broadly classified into three categories: elastic applications (data-oriented), tolerant real-time applications, and intolerant real-time applications.

Real-time applications as a class of applications need the data in each packet by a certain time and, if the data has not arrived by then, the data is essentially worthless, and elastic application as a class of applications will always wait for data to arrive. Elastic applications are applications that built on top of TCP protocol. TCP is a reliable transfer protocol that uses acknowledgements users working with applications based on symbolic data that can tolerate significant delays and loss. Such applications are considered non-real-time applications (NRT) that do not have stringent timing requirements and do not need any assurance from the network [75].

Data-oriented traffic can be further classified into (i) NRT asymmetric (NRTA) in which the requests are considerably using less resources than responses, such category can be further classified into interactive class and bulk-transfer class. Interactive class is suited for applications that use short packets, such as Telnet, web browsing, and enhanced web browsing. Bulk-transfer class is suited for store and forward applications that uses long packets, such as SMTP and FTP, and (ii) NRT symmetric (NRTS) in which requests and responses use the same amount of resources such as Internet chatting applications [76].

On the other hand, real-time applications (media-oriented) inherently have more stringent QoS requirements due to the nature of real-time transmissions. To achieve user satisfaction, the transmission infrastructure should strongly considers delay and jitter requirements to maintain system timing and constant data rate. Real-time applications can be divided into two broad categories: (i) tolerant real-time asymmetric applications (TRTA) are real-time applications that are very sensitive to delay bounds. Timeliness is very important for these real-time applications.

These applications can tolerate moderate end-to-end delay, so it is called soft real time. However, it requires high throughput and very low error rate. Common TRTA applications include those which are conversational in nature, such as multimedia conferencing that includes video conferencing, group of participants in teleconferencing audio, and audiographics conferencing that enables participants to share workspace and telephony service that involves videophone conferencing and VOIP, and (ii) intolerant real-time asymmetric applications (IRTA) demand more stringent QoS from the network. Such applications must have precise bandwidth, delay, and jitter constraints, and if the timing constraints are not met, such applications suffer from high performance degradation so it is called hard real time. Common IRTS applications are audio and video broadcasting, interactive audio, and video on demand and streaming media.

TRTA and IRTS applications are built on top of UDP protocol. UDP is unreliable protocol that does not have acknowledgment or flow control. So, such applications need more concern in QoS scope. QoS has three attributes to measure the output performance of a process: timeliness, precision, and accuracy. Timeliness measures the time taken to produce the output of the process. Precision measures the amount or quantity of the produced output. Accuracy measures the correctness of the produced output.

### 6.2. DSCP Assignation

By following the classification proposed in [25], some DSCP values may be dedicated to administrative and control traffic. Per hop behaviors (PHB) mapped by a codepoint with a larger numerical value should receive better or equal forwarding treatment than the one with the lowest numerical value. Respecting these guidelines, DSCP assignation is reported in Table 2, together with possible examples of traffic types and possible ranges of QoS performance parameters. The selected path should be calculated based on each application of QoS metric's requirement.

## 7. Routing Computation Framework

As reviewed in Section 4.3, the previously proposed algorithms (OQRA and PQRA) suffer from excessive computational complexities or low performance. This section presents a routing computation framework that proposes network diversities, by applying PQRA and OQRA routing paradigms into a real network, with low-computational complexity and high routing performance. In that sense we use dynamic programming (DP) technique. DP solves a sequence of larger and larger instances, reusing the previously saved solutions for the smaller instances, until a solution is obtained for the given instance. This simple idea can sometimes transform exponential-time algorithms into polynomial-time algorithms. Unlike the majority of routing algorithms, which assume an adjacency-list representation of the graph, most of the algorithms which rely on DP use an adjacency matrix representation. The adjacency matrix is a memory efficient way of representing dense graphs while linked list is more efficient for sparse graph [77].

The basic idea behind the proposed routing framework addresses the following issues: firstly, how to find all precomputed pairs of the shortest paths with two additive constraints and, secondly, how on-demand routing computation takes place when new requests arrive. The main idea is explored in two-phase framework.

In the first phase, QoS optimal paths precomputation routing algorithm (QOPRA), recursively, computes all-pairs-shortest paths in a graph of nodes, as presented in [78], connected in the forward direction according to *w*_1_ and in the backward direction according to *w*_2_ (Algorithm 1).

Each node precomputes the routing from itself into a destination prior to receiving the requests and stores this information in its FIT. The precomputation algorithm overhead is shared between different requests. Then, an on-demand routing algorithm (OQRA) (Algorithm 2) activates when there is an incoming request with QoS requirements to find the path that satisfies QoS constraints from these precomputed paths. Actually, proposed hybrid form can provide enough information to support efficient admission control, as well as less on-line computation overhead and high success rate.

### 7.1. Related Notations and Problem Analysis

For QoS assurance, we have to study all the subpaths between every pair of vertices in the graph instead of working with a single source to obtain more visibility and accurate path computation. However, this can be solved by repeating single source algorithm once for each vertex in the graph, but it requires more computations and incorporates more complexity.

Undirected graphs can be transformed into directed graphs, by replacing the undirected link with two directed links each assigned one weight. Using two generic nodes, labeled as nodes *x* and *y*, in a network of *N* nodes. Notations are as follows. Let *d*_*xy*_^*p*^ denote the length of the shortest path from vertex *x* to vertex *y*, where only the first *p* vertices are allowed to be intermediate vertices. If no such path exists, then let *d*_*xy*_^*p*^ = *∞*. From this definition of *d*_*xy*_^*p*^ it follows that *d*_*xy*_^0^ denotes the length of the shortest path from *x* to *y* that uses no intermediate vertices (i.e., directly connected). So, *d*_*xy*_^0^ = *∞* if the nodes are not directly connected; otherwise, *d*_*xy*_^0^ has a finite value and *d*_*xx*_^0^ = 0 for all vertices *x*. We have to rely on intermediate nodes and, accordingly, consider a node labeled *p* as intermediate between node *x* and node *y*.

**Figure 6:**
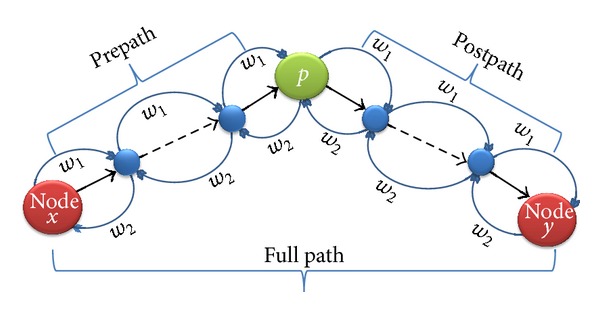
Finding MCSP from source *x* to destination *y*.

As presented in Figure 6, for finding multi-dimensional shortest path from node *x* to *y* assume that we know: (i) a shortest path from vertex *x* to vertex *y* that allows *p* vertices as intermediate vertices according to weight (*w*_1_) denoted as *d*_*xy*_^*p*,*w*_1_^, and (ii) a shortest path from vertex *y* to vertex *x* that allows *p* vertices as intermediate vertices according to weight (*w*_2_) denoted as *d*_*yx*_^*p*,*w*_2_^. The two terms in the minimum operator (2) identify that either node *p* is on the shortest path from nodes *x* to *y* or not:

(2) $$\begin{array}{c} d_{xy}^{p}=\min\left\{d_{xp}^{p-1}+d_{py}^{p-1},d_{xy}^{p-1}\right\},\\ d_{yx}^{p}=\min\left\{d_{yp}^{p-1}+d_{px}^{p-1},d_{yx}^{p-1}\right\}. \end{array}$$

Furthermore, *d*_*xy*_^*m*^ and *d*_*yx*_^*m*^ represent the length of the shortest path from *x* to *y* in the last iteration according to weights (*w*_1_) and (*w*_2_), respectively. Ultimately, we wish to determine *D* ^*m*^, the matrix of the shortest path lengths *d*_*xy*_^*m*^. The shortest path algorithm starts with *D* ^0^ and calculates *D* ^1^ from *D* ^0^ and then *D* ^2^ from *D* ^1^. This process is repeated until *D* ^*m*^ (the shortest path matrix) is calculated from *D* ^*m*−1^ using formula (2).

Formula (2) computes only one path between the nodes. It is beneficial to know the second or third shortest paths between two nodes. In order to compute *k*-shortest path, we have to execute a sequence of the arithmetic operations, namely, addition and minimization presented in [79]. Path lengths are now represented by a *k*-dimensional vector, *d*_*i*_ ∈ *R* ^*k*^.

Let *a* = [*a*_1_, *a*_2_,…, *a*_*k*_] and *b* = [*b*_1_, *b*_2_,…, *b*_*k*_] be members of *R* ^*k*^. Generalized minimization denoted by + and generalized addition denoted by × are defined as follows:

(3) $$\begin{array}{c} a+b=\underset{k}{\min}\left\{a_{i},b_{i}\;|\;i=1,2,3,\ldots ,k\right\},\\ a\times b=\underset{k}{\min}\left\{a_{i}+b_{i}\;|\;i=1,2,3,\ldots ,k\right\}. \end{array}$$

To retrieve multiple paths, utilizing (3) and (2) can be replaced by the following equation:

(4) $$\begin{array}{c} d_{xp}^{p}=d_{\mathrm{xp}}^{p}\times d_{xy}^{p}+d_{xy}^{p-1}. \end{array}$$

The problem is to obtain path(s) from a source *s* to destination *d* on *G* that satisfies multiple QoS constraints *L*_*i*_, where *i* = 1,2,…, *m*. QoS routing problem can have different definitions in the literature as follows.

**Definition 1 .**

In multiconstrained feasible path (MCFP) problem, find a feasible path (*P*) from *s* to *d* such that

(5) $$\begin{array}{c} w_{i}\left(P\right)=\underset{(x,y)\in P}{\sum }w_{i}\left(x,y\right)\leq L_{i}. \end{array}$$

**Definition 2 .**

In multiconstrained optimal path (MC (O) P) problem, find all feasible paths from *s* to *d* satisfying (2) and in addition minimize some length function *l*(*p*) such that *l*(*p*) ≤ *l*(*p* ^■^), for all feasible paths *p* ^■^ between *s* and *d* that satisfy MCFP.

**Definition 3 .**

In multiple-constrained shortest path (MCSP) problem, find optimal constrained shortest path (CSP) from *s* to *d* that obeys constraints and has the smallest hop count.

**Definition 4 .**

In *K*-multiple constrained shortest path (KMCSP) problem, find *K* feasible constrained shortest path from *s* to *d* subject to multiple constraints, and list them in order of increasing length, where *K* is the number of paths.

Definitions 2 and 4 need path length *l*(*P*) to be able to compare paths. The first choice was a linear path length; Jaffe proposed to use the next definition [32]:

(6) $$\begin{array}{c} l\left(P\right)=\overset{m}{\underset{i=1}{\sum }}d_{i}w_{i}\left(P\right)\;\text{where}\;\;d_{i}>0. \end{array}$$

**Figure 7:**
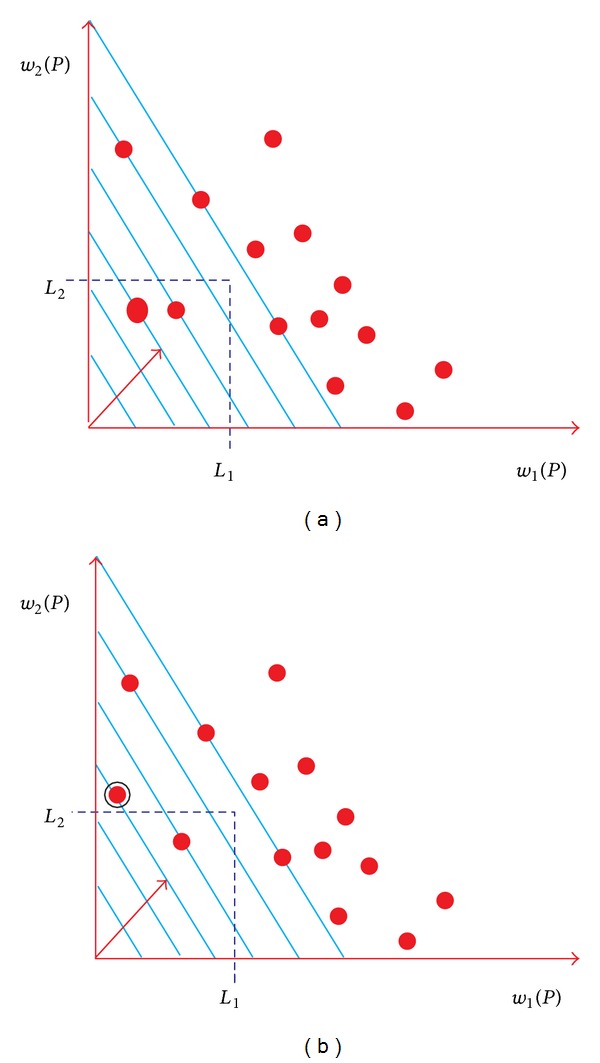
Using a linear path length, searching for a solution starts from origin until it hits a point: (a) algorithm succeeds to obtain a solution. (b) The algorithm fails as it finds solution outside constrained area.

The main advantage of linear path length algorithms is that by replacing each link vector via (6) by a single parameter, Dijkstra's algorithm can be deployed and so it is easy to develop a polynomial-time algorithm that minimizes *l*(*P*). Unfortunately, their major drawback, as Figure 7 depicts, is that (i) the shortest path returned by Dijkstra's algorithm is the first solution intersected by a set of parallel lines which may be infeasible (i.e., path outside the feasible region) and (ii) the area scanned outside the constrained area is considered large. Thus, Dijkstra's algorithm will not always work satisfactorily.

To overcome drawbacks of relying on linear path length, Van Mieghem and Kuipers noticed that the area scanned outside the constraint area can be further reduced if the straight equilength lines are replaced by curved equilength lines that more closely approach the boundary of the constraint area [39]. Therefore, they recommended the deployment of nonlinear representation of path length. Normalized nonlinear cost function for any path from the source to the destination is given as follows:

(7) $$\begin{array}{c} l\left(p\right)={\left(\frac{w_{1}\left(p\right)}{l_{1}}\right)}^{q}+{\left(\frac{w_{2}\left(p\right)}{l_{2}}\right)}^{q}+\cdots +{\left(\frac{w_{K}\left(p\right)}{l_{m}}\right)}^{q}, \end{array}$$

where *q* ≥ 1.

**Figure 8:**
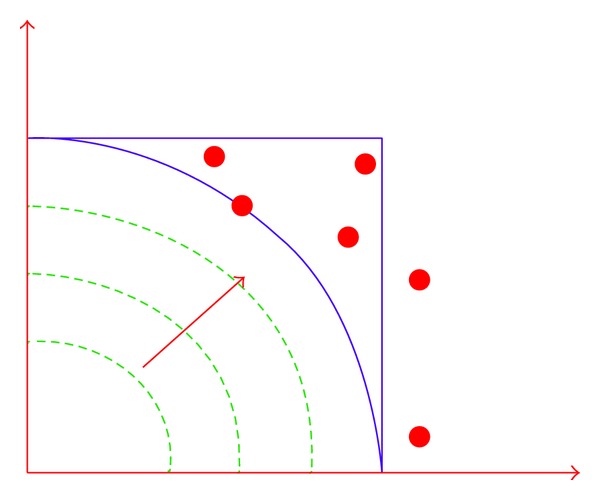
Using a nonlinear path length.

As Figure 8 depicts, feasible region can be scanned precisely. As *q* increases, the likelihood of finding a feasible path also increases. Therefore, to increase the probability of finding a feasible path, set *q* to *∞* and use the following cost function for a path [34]:

(8) $$\begin{array}{c} l\left(p\right)=\max\left\{\frac{w_{1}\left(p\right)}{l_{1}},\frac{w_{2}\left(p\right)}{l_{2}},\ldots ,\frac{w_{K}\left(p\right)}{l_{m}}\right\}. \end{array}$$

The length function (7) considers the value of the most critical constraint of a path regarding the end-to-end requirements. Nonlinear length algorithms are likely to outperform linear length algorithms.

Thus, the shortest path from vertex *x* to vertex *y* according to weight (*w*_1_) and weight (*w*_2_) can be given as follows:

(9) $$\begin{array}{c} \delta \left(x,y\right)=\max\left(\frac{d_{xy}^{m}}{l_{a}},\frac{d_{yx}^{m}}{l_{b}}\right). \end{array}$$

### 7.2. Metacode

Metacode of QOPRA starts with initialization. The module INITIALIZE initializes the necessary parameters for the main algorithm (Algorithm 1). Determine the matrix *D* ^0^ whose *xy*th elements equal the length of the shortest arc according to *w*_1_ from vertex *x* to vertex *y*, if any. The *yx*th element equals the length of the shortest arc from vertex *x* to vertex *y* according to *w*_2_. If no such arc exists, let *d*_*xy*_^0^ = *∞*. Let *d*_*xx*_^0^ = 0. The actual arcs that comprise each shortest path are also recorded in the *π* matrix from which we obtain tentative along the shortest paths. The INITIALIZE module sets *π* matrix as follows: if there is no direct link between nodes *x* and *y*, it sets *π* ^0^(*x*, *y*) to Nill. If there is direct link from nodes *x* to *y*, it sets *π* ^0^(*x*, *y*) to *x* and sets *π* ^0^(*x*, *x*) to *x*.

QOPRA successively determines the elements of *D* ^1^ from the elements of *D* ^0^ and the elements of *D* ^2^ from *D* ^1^ until obtaining *D* ^*M*^ from *D* ^*M*−1^ using the recursive formula (5). As each element *D*_*xy*_^*p*^ is determined, record the corresponding path through the computation of matrix *π* ^*p*^. We need only to record one vertex for *P*_*xy*_^*p*^. If *P*_*xy*_^*p*^ is known for all vertices *x* and *y*, then all the vertices along the shortest path from *x* to *y* can be found as follows: set *P*_*xy*_^*p*^ equal to *x* for all *y*. Do this for all vertices *x*. Then, as the algorithm is performed, whenever the minimum on the left side of (9) is the first term, set *P*_*xy*_^*p*^ equal to *P*_*py*_^*p*^. Otherwise, leave *P*_*xy*_^*p*^ unchanged.

Upon termination of the algorithm, the *xy*th element of matrix *D* ^*M*^ represents the length of the shortest path from vertex *x* to vertex *y* according to *w*_1_ and the *yx*th element of matrix *D* ^*M*^ represents the length of the shortest path from vertex *x* to vertex *y* according to *w*_2_. Also the *π* ^*M*^ matrix contains the next-to-last vertex in that path.

The module OQRA (Algorithm 2) calculates the shortest path using nonlinear path length (4) from a source node *x* to all nodes using matrix *D* ^*M*^ evaluated from the module QOPRA. The shortest path from vertex *x* to vertex *y* according to weight (*w*_1_) and weight (*w*_2_) can be obtained using (6).

OQRA gets the path that satisfies the length function (6) according to constraints (*l*_*a*_, *l*_*b*_) and prints the corresponding path in a vector called Path[] and its hop count (HC) by calling the PrintPath module. If there is more than one path with the same length, it retrieves the one with the least hop count by calling the PrintPath module which returns the paths and the hop count HC1 corresponding to Path 1 and HC2 corresponding to Path 1.

OQRA procedure obtains the shortest path *δ*_(*x*,*y*)_ and the nodes on that path by calling the procedure OQRA and *π* ^*M*^ matrix. Obtaining the nodes on the shortest path can be achieved by calling the module PrintPath (Algorithm 3).

We often wish to compute not only the shortest path length, but also the vertices on the shortest path as well. The module PrintPath returns the nodes on the best path by using matrix *π* ^*M*^ evaluated from the module QOPRA. It gets the path by making a recursion calling to itself until the full path is obtained. It also returns the hop count of that path. It starts with an empty vector called PathVector and checks the *π* ^*M*^ matrix for the path from *x* to *y* as follows: it sets *p* = *π*_*xy*_^*M*^ and adds *k* to the PathVector and makes a recursive calling to itself until it reaches the source *x* in the *π* ^*M*^ matrix.

## 8. Proposed QMPF Model

To implement proportional differentiation, the majority of related work proposed priority-based scheduling (PS) algorithms. PS enforces proportional delay differentiation by dynamically adjusting the priority of a given class as a function of the waiting time experienced by packets from that class [80, 81]. Alternatively, rate-based schedulers provide proportional differentiation by dynamically changing the service rates allocated to classes [82]. Here, we provide a slightly different approach. Instead of relying on actual waiting time experienced by each packet, we deploy the proportional model in the differentiation of average class queuing delay to define class priority and investigate appropriate packet scheduling mechanisms (Algorithm 4).

**Figure 9:**
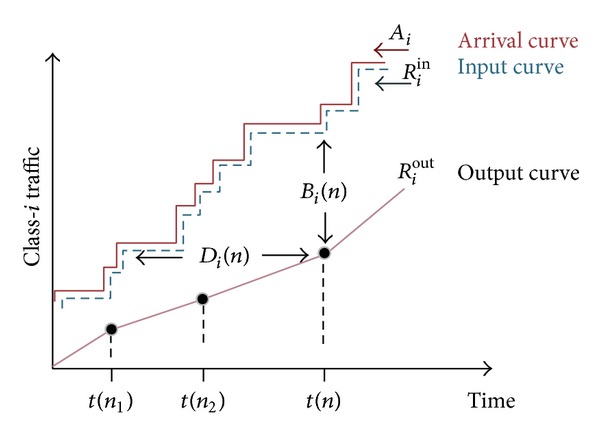
Concepts of arrival curve, input curve, and output curve for class *i* traffic.

Average class queuing delay is determined by the arrival rate of packets, the amount of traffic removed from the queue, and other service classes (queues). We start by deriving the average queuing delay for each class. The derivation draws inspiration from [80–84]. In Figure 9, the concepts of arrival curve, input curve, and output curve for class *i* traffic are depicted.

### 8.1. Average Queuing Delay per Class

Consider a discrete, event-driven time model, where events are traffic arrivals with the following notations:

- *t*(*n*): the time of the *n*th event,
- Δ*t*(*n*): the time elapsed between the *n*th and the (*n* + 1)th events,
- *λ*_*i*_(*n*): the class *i* arrivals at *n*th event,
- *χ*_*i*_(*n*): the quantum allocated to class *i* at the time of the *n*th event. The quantum is the amount of bytes that class *i* could transmit if it is selected for transmission; otherwise, it is set to zero.

The arrival curve for class *i* at the *n*th event, *A*_*i*_(*n*), is the total traffic that has arrived to the transmission queue of class *i* since the beginning of the current busy period; that is,

(10) $$\begin{array}{c} A_{i}\left(n\right)=\overset{n}{\underset{k=0}{\sum }}\lambda_{i}\left(k\right). \end{array}$$

Assuming that queuing model is lossless and that there are large enough buffers for packets that need to be queued and hence no traffic is dropped, then the input curve, *R*_*i*_^in^, is the traffic that has been entered into the transmission queue at the *n*th event that equals *A*_*i*_(*n*):

(11) $$\begin{array}{c} R_{i}^{\text{in}}=A_{i}\left(n\right). \end{array}$$

The output curve is the traffic that has been transmitted since the beginning of the current busy period; that is,

(12) $$\begin{array}{c} R_{i}^{\text{out}}=\overset{n}{\underset{k=0}{\sum }}\chi_{i}\left(k\right)\Delta t\left(n\right). \end{array}$$

For event *n*, the vertical distance between the input and output curves denotes the class *i* backlog *B*_*i*_(*n*) and the and horizontal distance denotes class *i* delay *D*_*i*_(*n*). For the *n*th event, we have

(13) $$\begin{array}{c} B_{i}\left(n\right)=R_{i}^{\text{in}}-R_{i}^{\text{out}},\\ D_{i}\left(n\right)=t\left(n\right)-t\left(\sup\left\{k<n:R_{i}^{\text{in}}\leq R_{i}^{\text{out}}\right\}\right). \end{array}$$

The minimum average delay $\bar{d_{i}}\left(n\right)$ for all the packets that have already arrived to class *i* can be calculated as

(14) $$\begin{array}{c} \bar{d_{i}}\left(n\right)=\frac{D_{i}\left(n\right)+B_{i}\left(n\right)}{\chi_{i}\left(n\right)}. \end{array}$$

At time *n*, the scheduler calculates for all classes (for transmission), the minimum possible normalized average delay, then selects the class with the maximum normalized average delay. That is, the order in which classes are selected for service is determined by the C-PDR-DWWR scheduler according to the following priority function:

(15) $$\begin{array}{c} j=\underset{i}{\max}\;\overset{-}{d_{i}}\left(n\right)\delta_{i}. \end{array}$$

So, high priority queues are serviced first such that the average delay experienced by packets in a delay class is inversely proportional to the delay weight of the class.

Within a selected class, queues are serviced in DWRR fashion. The main objective is to provide accurate control over the amount of data (i.e., quantum of service (*χ*)) sent to the path selected by path selection component. The quantum of service (*χ*) is proportional to path bandwidth and output buffer size. The quantum of service (*χ*) is calculated such that maximum end-to-end delay can be minimized. The deficit counter (DC) specifies the total number of bytes that the queue is permitted to transmit. The DC of a queue is incremented by a quantum (*χ*) each time the queue is visited by the scheduler.

### 8.2. End-to-End Delay and Service Quantum Computation

Nodes (vertices) will be labeled with the generic label *v* (*v* = 1,2,…, *V*), links (edges) with label *a* (*a* = 1,2,…, *A*), and flows within a service class with label *f* (*f* = 1,2,…, *F*). The capacity of link *a* will be denoted by *c*_*a*_. Each flow *f* is characterized by the flow volume denoted by *h*_*f*_. For flow *f* the total number of assigned paths is denoted by *P*_*f*_ and they are labeled with *p* from the first path to the total number of paths; that is *p* = 1,2,…, *P*_*f*_; this sequence is called the list of candidate paths.

To tie it to generic flow *f*,we write the list of paths as *Pf* = (*Pf*1, *Pf*2,…, *Pf* *Pf*). Flow volumes are realized by means of flows assigned to paths on their routing lists. The flow realizing flow *f* on path *p* is denoted by *χfp* (*p* = 1,2,…, *Pd*). Suppose that we denote the vector of flows assigned to flow *f* with *χf* = (*xf*1, *xf*2,…, *xf* *Pf*) for path indices *p* = 1,2,…, *Pf*; then we arrive at

(16) $$\begin{array}{c} \chi f1+\chi f2+\cdots +\chi fPf=hf. \end{array}$$

In summation notation, we can write this as

(17) $$\begin{array}{c} \underset{p}{\sum }\chi_{fp}=h_{f}\;f=1,2,3,\ldots F. \end{array}$$

In general, the vector of all flows (path-flow variables) will be called flow allocation vector or simply flow vector, which can be written as

(18) χ=(χ1,χ2,…,χf)=(x11,x12,…,x1P1,x21,x22,…,x2P2,…,xf1,xf2,…,xfPf)=(xfp:d=1,2,…,f;p=1,2,…,Pf).

Assume firstly that flow arrival follows the Poisson process and that the flow size is exponentially distributed. The system can be thought of as the famous *M*/*M*/1 queueing system in which arrival rate *λ* and the service rate *μ* are the same. If the average size of a flow assigned to a link is denoted by *χ* bits, then the average service rate and arrival rate of the link are

(19) $$\begin{array}{c} \mu_{a}=\frac{c_{a}}{\chi },\\ \lambda_{a}=\frac{l_{a}}{\chi }. \end{array}$$

Minimizing average path delay being weighted by its corresponding traffic of all paths between source and destination pairs is essential. Path delay is the time it takes a packet to travel across the network from one end to the other end.

Efficient QoS LDM must provide well-tailored load balancing and preserve packet ordering. Well load balancing can be achieved via assigning load on each path properly with respect to path bandwidth and buffer size. The packet ordering is likely to increase in a network with a large degree of parallelism. Assigning packets to different paths which have the same delay leads to increasing the probability of packet ordering preserving.

Thus, minimizing average path delay is crucial and involves minimizing link delay for each link that belongs to the path. The link delay is composed of two components, namely, propagation delay and queuing delay. Propagation delay *D*_*a*_ is a fixed value, whereas, queuing delay (*Q*_*a*_) varies according to the input traffic rate (*λ*), the bandwidth capacity of the path (*μ*_*p*_), and the traffic splitting ratio (*χ*). Queuing delay can be decreased using load balancing. Thus, we can write

(20) $$\begin{array}{c} D_{p}=\underset{a\in p}{\sum }D_{l}+Q_{p},\\ Q_{p}=\underset{a\in p}{\sum }\frac{l_{a}}{c_{a}-l_{a}}. \end{array}$$

At the link level, we should minimize average queuing link delay. The average queuing link delay can be evaluated using the following function (*F*):

(21) $$\begin{array}{c} F=\frac{l_{a}}{c_{a}-l_{a}},\;\text{for}\;\;0\leq \frac{l_{a}}{c_{a}}<1. \end{array}$$

Function *F* is a nonlinear convex function which is discontinuous at *l*_*a*_ = *c*_*a*_. The good news is that it is possible to substitute convex mathematical programming problems with their piecewise linear approximations problems. Function *F* can be transformed into linear by using piecewise linear approximation presented by Fortz et al. [53–55]. Fortz and Throup proposed a six-segment piecewise linear cost function which is useful in tuning IGP metric that is where the routing cost for each arc is an increasing convex function of its utilization. Fortz and Throup function is given by

(22) $$\begin{array}{c} \dot{\varphi }=\{\begin{array}{cc} 1 & \text{for}\;\;0\leq \frac{l_{a}}{c_{a}}<\frac{1}{3}\\ 3 & \text{for}\;\;\frac{1}{3}\leq \frac{l_{a}}{c_{a}}<\frac{2}{3}\\ 10 & \text{for}\;\;\frac{2}{3}\leq \frac{l_{a}}{c_{a}}<\frac{9}{10}\\ 70 & \text{for}\;\;\frac{9}{10}\leq \frac{l_{a}}{c_{a}}<1\\ 500 & \text{for}\;\;1\leq \frac{l_{a}}{c_{a}}<\frac{11}{10}\\ 5000 & \text{for}\;\;\frac{11}{10}\leq \frac{l_{a}}{c_{a}}<\infty . \end{array} \end{array}$$

The Fortz and Throup function is a piecewise linear envelope of the load latency function, scaled by *c*. Thus, we can say that $c_{a}\cdot F\approx \dot{\varphi }$. For the objective of minimizing weighted average queuing path delay,

(23) $$\begin{array}{c} \text{Minimize}\;\;F\left(x\right)=\underset{a\in P}{\sum }\frac{\varphi \left(l_{a},c_{a}\right)}{c_{a}}, \end{array}$$

where

(24) $$\begin{array}{c} l_{a}=\lambda_{a}\cdot \chi ,\\ C_{a}=\mu_{a}\cdot \chi . \end{array}$$

Thus, we can write the end-to-end path delay considering *M*/*M*/1 queuing system that can be formulated as follows:

(25) $$\begin{array}{c} D_{p}\left(\chi \right)=\underset{a\in p}{\sum }D_{a}+\underset{a\in P}{\sum }\frac{\varphi \left(l_{a},c_{a}\right)}{c_{a}}. \end{array}$$

Formula (25) is designed for Poisson traffic and is thus likely not practical for a real network under different traffic conditions. As in [67], with the assumption that input traffic is a combination of Poisson traffic and unknown traffic, a third term is added to formula (25). The third term evaluates the waiting time of the current packet at an input queue. Formula (25) becomes

(26) $$\begin{array}{c} D_{p}\left(\chi \right)=\underset{a\in p}{\sum }D_{a}+\left(1-\omega \right)\underset{a\in P}{\sum }\frac{\varphi \left(l_{a},c_{a}\right)}{\mu_{a}\cdot \chi }+\omega \frac{q_{p}}{\mu_{p}}, \end{array}$$

where *ω* is weight factor that controls the weight between theoretical queuing delay and instantaneous queuing delay. *q*_*p*_ is the current queuing size of the buffer of each path *p*. The optimization problem can be formulated as follows:

(27) $$\begin{array}{c} \text{Minimize}\;\underset{p_{i}\in P}{\max}D_{p}\left(\chi \right) \end{array}$$

subject to

(28) $$\begin{array}{c} \underset{p}{\sum }\chi_{fp}=h_{f}\;f=1,2,3,\ldots ,F\\ \underset{p}{\sum }u_{fp}=K_{f}\;f=1,2,3,\ldots ,F\\ x_{fp}\leq u_{fp}h_{f}\;f=1,2,3,\ldots ,F \end{array}$$

constants:

  
$\delta_{efp}\{\begin{array}{cc} =1 & \text{if}\;\text{link}\;a\;\text{belongs}\;\text{to}\;\text{path}\;p\;\text{realizing}\;\text{flow}\;f\\ =0 & \text{Otherwise} \end{array}$
 
*h*_*f*_: volume of flow *f*
 
*K*_*f*_: predetermined number of paths for flow *f*

variables:

 
*χ*_*fp*_: quantum of flow allocated on path *p*
 
*u*_*fp*_: binary variable corresponding to the flow variable *f*_*dp*_.

## 9. Performance Evaluation

The aim of experiments presented in this section is to demonstrate the effectiveness of proposed DQARE architecture and prove that it is capable of overcoming OSPFxQoS limitations. During the experiments, a number of simulations were conducted using MATLAB and NS2 [85]. Firstly, OSPFxQoS and DQARE behaviors are evaluated. Secondly, the proposed routing algorithm and multipath forwarding model have to be evaluated with their counterparts in the literature.

### 9.1. OSPFxQoS and DQARE Architectures Evaluation

OSPF and DQARE behavior has been investigated for different scenarios built under network simulation tool (NS2) environment [85]. NS2 is a discrete event driven simulator which means that it starts packet sending at the designated time and stops also at a determined time. In this experiment, the OSPFxQoS environment has been firstly implemented. Then, we modify OSPFxQoS operation with our proposed priority treatment, QoS routing, and forwarding mechanisms. We created network topology with Gt/itm tool that exists in NS2 simulator. We have taken networks of 10, 25, 50, 100, 150, 200, and 250 nodes and simulation time = 300 sec in our scenario files. OSPF costs of the links are assigned randomly according to the guideline given in [5]. Also, each link delay is created randomly.

We generate cross traffic in all scenarios to account for the network traffic flowing through nodes. This cross traffic is generated as follows: source and destination nodes are randomly chosen. Then each source and destination pair exchange traffic, which follows a Poisson distribution. The elastic traffic and real-time traffic are created and delivered via this test network. As shown in Table 3, different parameters are settled; for example, TCP and UDP traffic are considered and, in all simulations, constant bit rate (CBR) is applied with intervals 0.02 ms.

#### 9.1.1. Performance Metrics

For performance comparison between DQARE and OSPFxQoS, we choose five key performance metrics, namely, the average end-to-end delay, packet delivery ratio, throughput, and control overhead.

*Average End-to-End Delay of Data Packets (AD).* Average time taken by a data packet to arrive at the destination include delays due to route acquisition, reservations, buffering and processing at intermediate nodes, and retransmission delays at the MAC layer. AD can be expressed mathematically as

(29) $$\begin{array}{c} \text{AD}=\overset{N}{\underset{i=1}{\sum }}\frac{T_{i}^{r}-T_{i}^{s}}{N}, \end{array}$$

where *T*_*i*_^*r*^ is receiving time of packet *i*, *T*_*i*_^*r*^ is sending time of packet *i*, and *N* is the number of connections. 

*Throughput (T).* The average of successful message delivery over a communication channel. It can be expressed mathematically as

(30) $$\begin{array}{c} T=\frac{N_{D}*S}{\text{TS}}\frac{b}{s}, \end{array}$$

where *N*_*D*_ is the number of delivered packets, *S* is packet size, and TS is the simulation time. 

*Packet Delivery Ratio (PDR)*. It is the ratio of number of data packets successfully received by hosts to the total number of data packets sent:

(31) $$\begin{array}{c} \text{PDR}=\frac{\sum \text{Number of packets recieved}}{\sum \text{Number}\;\text{of}\;\text{packets}\;\text{sent}}. \end{array}$$

*Control overhead* is the ratio of total number of routing control packets sent to describe the changes in the dynamic topology to the total number of data packets delivered successfully:

(32) $$\begin{array}{c} \text{Control}\;\text{overhead}=\frac{\sum \text{Routing}\;\text{control}\;\text{Packets}\;}{\sum \text{Data}\;\text{Packets}\;\text{recieved}\;}. \end{array}$$

#### 9.1.2. Results

**Figure 10:**
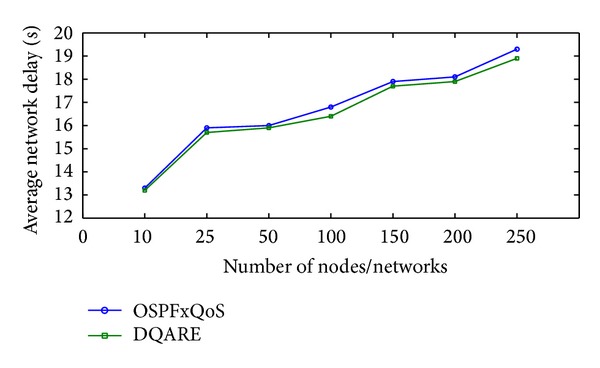
Average delay/network versus number of nodes.

(*1) Varying Number of Nodes.* In the first experiment, we measure the performance of OSPFxQoS and DQARE by varying the number of nodes as 10, 25, 50, 100, 150, 200, and 250.  Figure 10 depicts the performance comparison of end-to-end average delay in the network. DQARE almost outperforms OSPFxQoS. This is because OSPFxQoS used RVSP, while DQARE used priority treatment, classification, and scheduling. Actually, improvement in average end–to-end delay in DQARE results from the MPF model which makes use of multiple paths.

**Figure 11:**
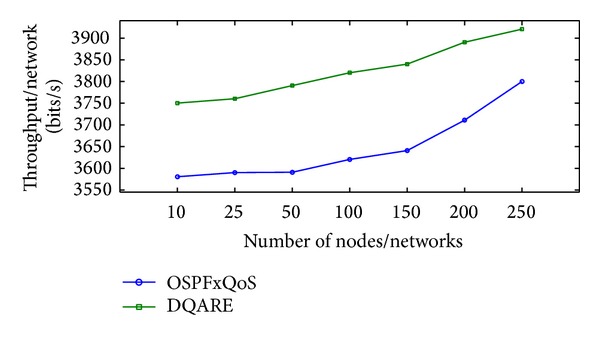
Average throughput/network versus number of nodes.

The difference of throughput between OSPF and DQARE environments, depicted in Figure 11, is quite obvious. OSPFxQoS gives single shortest paths based upon precomputation routing scheme. Retrieved paths may have common segments that become bottlenecked. When congestion occurs, OSPFxQoS cannot shift traffic to better alternative paths to mitigate congestion. On the other hand, DQARE architecture is supplied with QMPF model and path/congestion manger. Multiple paths exploitation is crucial for circumventing congestions scenarios. Also, if congestion occurs, path/congestion manager transfers quickly traffic flow from congested paths to another better path without severe loss of traffic.

**Figure 12:**
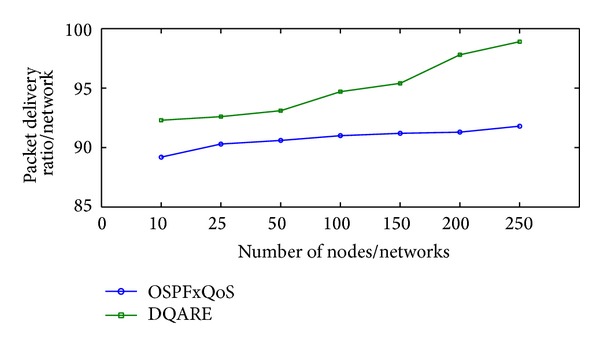
Packet delivery ratio/network versus number of nodes.

Figure 12 shows the packet delivery ratio of OSPFxQoS and DQARE with different network topologies. We can find that as the number of nodes/network increases packet delivery fraction in case of DQARE outperforms OSPFxQoS. This is because OSPFxQoS lacks self-adaptation mechanism. Routers disseminate information only when topology changes. OSPFxQoS is unable to readjust forwarding paths in order to lessen the impact of failures. Also, OSPFxQoS is unable to load-balance traffic to optimize the performance of the network. On the other hand, DQARE uses an adaptive route mechanism implemented via path/congestion manager and QoS load balancing module. Thus, DQARE significantly enhances the usage of network capacity.

**Figure 13:**
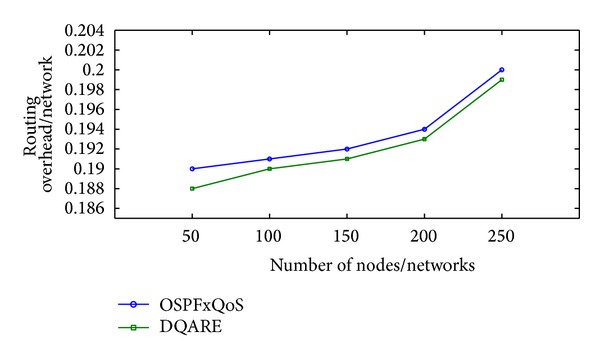
Control overhead/network versus number of nodes.

**Figure 14:**
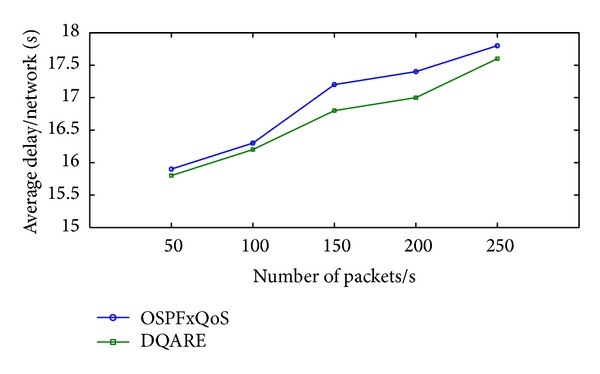
Average delay versus arrival rate.

**Figure 15:**
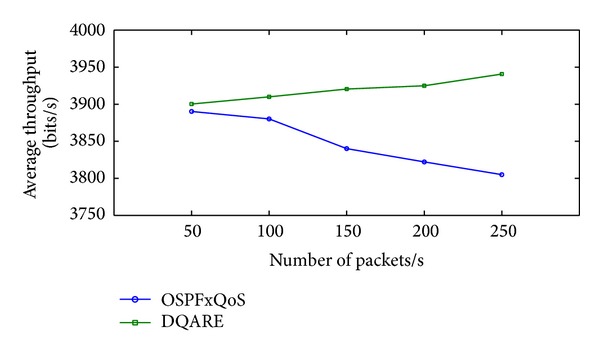
Throughput versus arrival rate.

**Figure 16:**
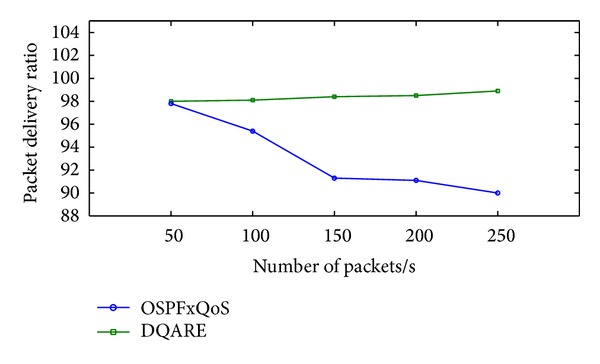
Packet delivery ratio versus arrival rate.

**Figure 17:**
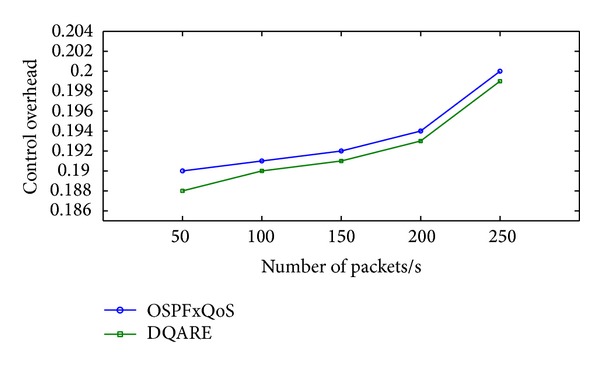
Control overhead versus arrival rate.

Figure 13 shows the performance comparison of routing overhead per network, and under this condition DQARE continues to outperform OSPFxQoS. Routing overhead in OSPFxQoS grows rapidly with the changes in the network topology; this is because OSPFxQoS deployed a precomputation routing algorithm routing that suffers from nonoptimal routing during congestion. OSPFxQoS needs to quickly initiate a new route discovery process when a link fails and therefore needs to consume a large amount of routing overhead. Our proposed routing algorithm retrieves multiple paths from a source to destinations and thus has the ability to use these multiple paths, so the overhead is smaller than OSPFxQoS.

(*2) Varying Packet Arrival Rate.* Figures 14, 15, 16, and 17 depict the behavior of both OSPFxQoS and DQARE for a network topology of 100 nodes. At each node the packet arrival rates are changed. Packet arrival rates are 50, 100, 150, 200, and 250 packet/sec.  Figure 14 depicts that DQARE outperforms OSPFxQoS in providing lower delay with the increasing arrival rate. The average throughput and delivery ratio gap between DQARE and OSPFxQoS increase along with arrival rate.  Figure 17 depicts the control overhead of DQARE architecture considered to be acceptable with the increasing in arrival rate.

### 9.2. Routing Algorithm Evaluation

Our first simulation is to show the intensity of the proposed routing algorithm by comparing the results with heuristics and exact algorithms in the literature. The proposed QoS routing algorithm is compared with RMCOP, HMCOP, SMACRA, HCA, and MPLMR algorithms coded in MATLAB 2012 and implemented on an Intel Core i3, 2.5 GHz CPU with 3 GB RAM running on Windows 7 professional.

#### 9.2.1. Network Topology

Network topologies used for simulations are randomly generated based on Waxman's model [86] with 50, 100, 200, 300, and 500 nodes. Waxman graph is considered counterpart to realistic telecommunication networks. In that sense, the location of nodes is randomly generated within the area of the graph. The probability of the existence of a link between nodes *x* and *y* is related to some function of the distance between these nodes. Formally, in Waxman graphs the probability (*p*_*xy*_) that two nodes *x* and *y* are connected equals $f\left(\vec{r_{x}}-\vec{r_{y}}\right)$, where $\vec{r_{x}}$ and $\vec{r_{y}}$ represent the position of node *x* and node *y*, respectively. So the farther the distance between two nodes, the smaller the need for a direct link between them. The probability function of Waxman's model is as follows:

(33) $$\begin{array}{c} p_{xy}=\alpha \cdot \exp\left[\frac{-\delta \left(x,y\right)}{\beta L}\right], \end{array}$$

where *α* represents the maximum link probability, *β* represents the parameter to control the link length, *L* is the maximum distance between two nodes in the graph, and *δ*(*x*, *y*) is the distance between *x* and *y*. In experiments, we set *α* = 0.8 and *β* = 0.9.

#### 9.2.2. Performance Metrics

We contrast the performance of various path selection algorithms using complexity and success rate (SR). Low complexity is the main goal of multiconstrained QoS routing schemes. A significant performance measure for the complexity of routing algorithms is time complexity in terms of execution time (ET). SR is the fraction of connection requests for which a feasible path is found. During all simulations, the success rate and execution time were stored.

#### 9.2.3. Simulation Model and Performance Measures

After generating graphs, we associate two randomly generated additive weights with each link (*i*, *j*). These weights are selected from uniform distribution sets. These weights are assigned, as depicted in Table 4, according to two types of correlation between them. No correlation assumes that both weights are independently selected from one set. While negative correlation assumes that one of the weights is selected from a set with small mean, the other is selected from another set with large mean.

In each run, source and destination nodes and QoS requirements of a request are randomly generated. The results reported in the subsequent sections are averaged over several runs. In each run, 10 random graphs are generated. For each random graph, ten independent link weights are generated using different random seeds. Each graph is subjected to 10 requests with different QoS constraints. There are about 1000 to 3000 connection requests that are generated for graphs with 50, 100, 200, 300, 400, and 500 nodes, respectively.

#### 9.2.4. Results

**Figure 18:**
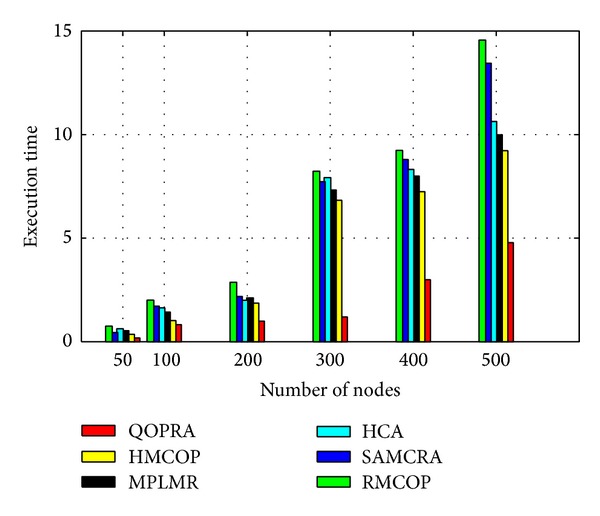
ET using uncorrelated link weights.

**Figure 19:**
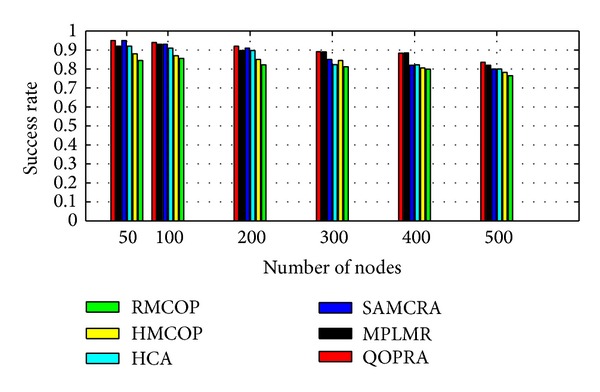
SR using uncorrelated link weights.

Using extensive simulations on the Waxman random graph with uncorrelated link weights, Figures 18 and 19 show that under the same level of computational complexity proposed algorithms (QOPRA) outperforms its contenders in its success rate and computational complexity.

If the primary cost of HMCOP is not available it finds only a feasible path and postpath which may misguide the selection of prepath. The performance of HMCOP in finding feasible paths can be improved by using the *k*-shortest path algorithm and by eliminating dominated paths. SAMCRA worst case complexity grows exponentially and it may be subject to some error decision rate. The absolute complexity of HCA is greater than SAMCRA complexity. HCA stops at the first feasible path it finds, so it has low execution time. RMCOP algorithm suffers from high complexity. MPLMR time complexity is comparable to HMCOP. However, MPLMR has a higher success rate than HMCOP.

**Figure 20:**
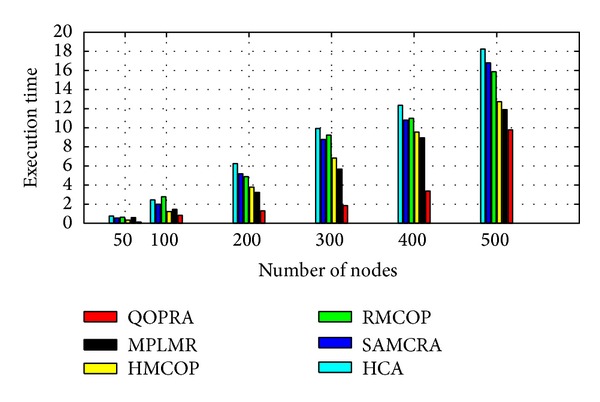
ET using negative correlated link weights.

**Figure 21:**
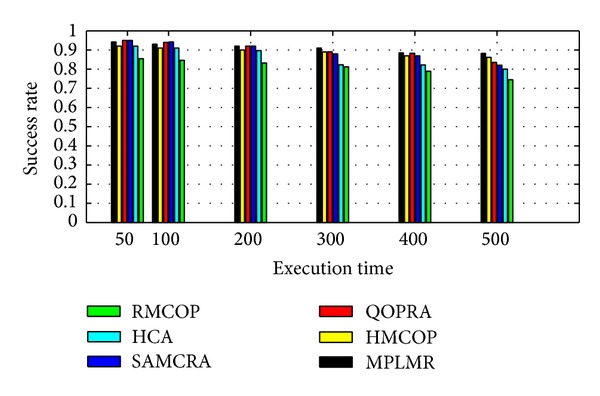
SR using negative correlated link weights.

As Figures 20 and 21 depict, using negatively correlated link weights, QOPRA still outperforms other algorithms. Negatively correlated link weights result in more paths in the network for which *w*_1_(*P*) ≫ *w*_2_(*P*) and vice versa. This situation degrades the performance of MPLMR and HLA. Also, in such case algorithms such as SAMCRA incur a large execution time with the increase in node number. However, SAMCRA achieves a higher success rate because it used path dominance and look-ahead techniques.

### 9.3. MPF Algorithm Evaluation

In this section, extensive network simulations are conducted to evaluate the performance of multipath forwarding mechanisms. We analyze the performance of proposed QMPF. FLARE, EDCLD, and CBM models are used for comparisons.

#### 9.3.1. Performance Metrics

Simulation-based verifications are presented in terms of (i) end-to-end delay which can be defined as the sum of propagation delay and queuing delay as defined in (26), (ii) Jitter, variation of end-to-end packet delay, and (iii) total packet delay which is the sum of end-to-end delay and packet reordering recovery delay.

#### 9.3.2. Simulation Method

**Figure 22:**
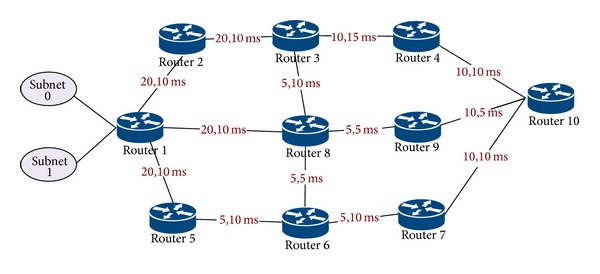
Simulated network topology.

Our simulations are based on NS2 [85].  Figure 22 shows the network topology adopted for the simulations. Each link is assigned with bandwidth and fixed propagation delay. The buffer size of router is set to 220 packets. TCP traffic and UDP traffic are generated from two subnets, 0 and 1, destined for node 10. Each subnet represented 50 traffic-generating hosts.

**Figure 23:**
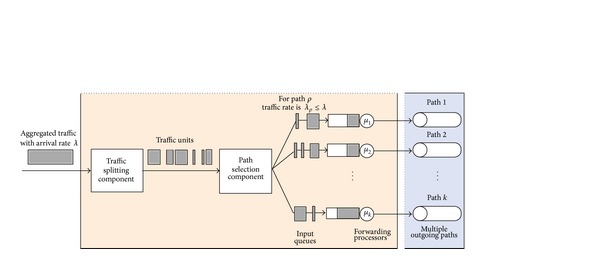
Data-plane extensions for MPF.

Each router is supplied with multiple paths to node 10. MPF mechanism is conducted under the environment shown in Figure 23. The input traffic to each node from 1 to 10 will be split into available paths. Load condition varies from low to high. The mean service time is inversely proportional to the bandwidth capacity (1/*μ*). The parameter *λ* is proportional to the total bandwidth of the paths. The mean packet arrival rate is chosen such that the ratio of the mean offered load to the mean service rate *λ*/*μ* varies from 0.1 to 0.9 with a step size of 0.1. Parameter *ω* in (23) is chosen to be 0.5.

#### 9.3.3. Simulation Results

**Figure 24:**
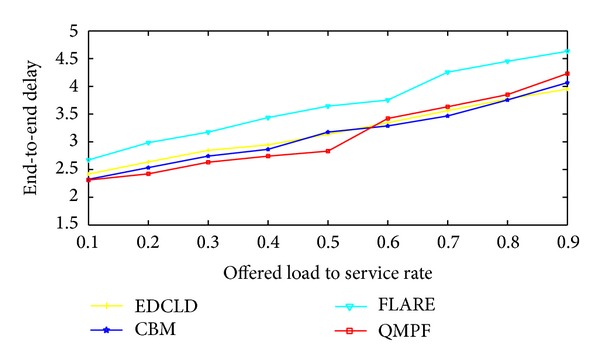
End-to-end packet delay.

Figure 24 compares the mean of end-to end delay achieved by various LDMs. As the ratio of input rate to output rate (i.e., *λ*/*μ*) increased, the mean value of total packet delay rises as well. For low to medium load QMPF achieves the least end-to-end delay. When the traffic load becomes heavier, the performance of QMPF degrades due to classification and scheduling overhead. Figure 24 also shows that CBM and EDCLD achieve near results. However, CBM has a smaller end-to-end delay. FLARE achieves the largest end-to-end delay due to load imbalance, especially at the high packet arrival rate.

**Figure 25:**
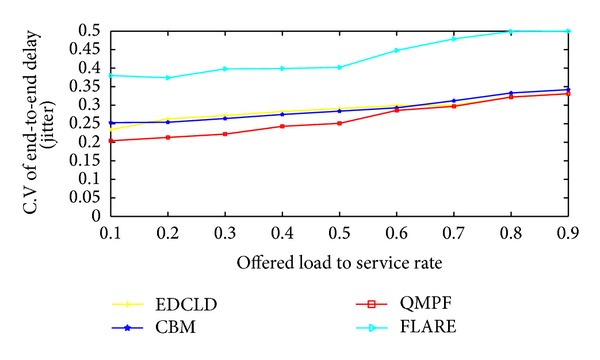
Coefficient variation of end-to-end packet delay.

Packet delay variation is depicted in Figure 25. The relationship between coefficient variation (CV) of end-to-end packet delay and the ratio of offered load to service rate is constructed. A large CV indicates a high risk of packet reordering. In light load QMPF achieves the least delay variation. As the ratio of input rate to output rate increases, CBM and EDCLD outperform QMPF.

**Figure 26:**
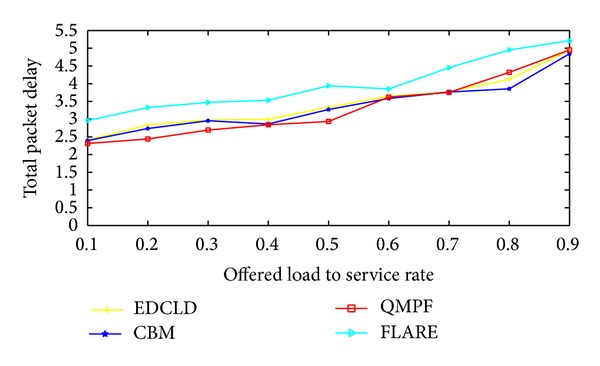
Total packet delay.

The total packet delay is an important indicator for QoS-oriented application. QMPF, CBM, and E-DCLD aim to decrease end-to-end delay and packet reordering delay and can thus efficiently reduce the total packet delay.  Figure 26 indicates that when the ratio (*λ*/*μ*) is larger than 0.6, the total packet delay counted by QMPF is slightly larger than E-DCLD and CBM.

## 10. Conclusion

QoS routing plays an important role in QoS provisioning. This paper introduced a generic distributed QoS adaptive routing engine (DQARE) architecture based on OSPFxQoS. DQARE architecture is furnished with three relevant traffic control schemes that shape the design of a QoS solution, namely, service differentiation, QoS routing, and QoS intradomain traffic engineering (TE).

Accordingly, this paper provided a general configuration guideline for service differentiation. Also, this paper introduced a QoS routing algorithm (QOPRA), based on dynamic programming technique. QOPRA attempts to obtain the optimal multiple paths in terms of two additive metrics. This objective is proved in the proposed work with minimal complexity and low error decision rate. This paper also proposed a new effective QoS* load distribution* model (QMPF). QMPF aimed to efficiently utilize multiple available paths and minimize the difference among end-to-end delays, jitter, and packet reordering. NS2-based simulations proved DQARE superiority over OSPFxQoS.

## Figures and Tables

**Algorithm 1:**
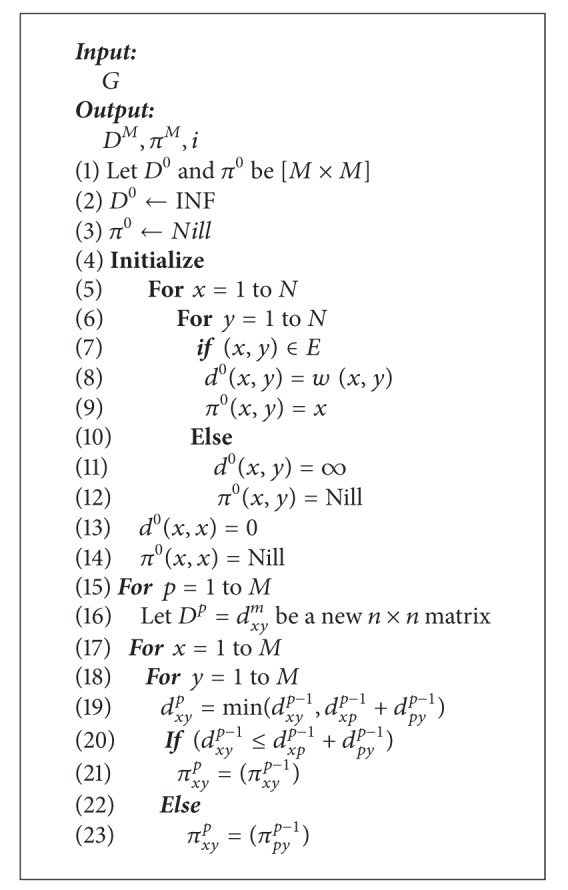
QOPRA.

**Algorithm 2:**
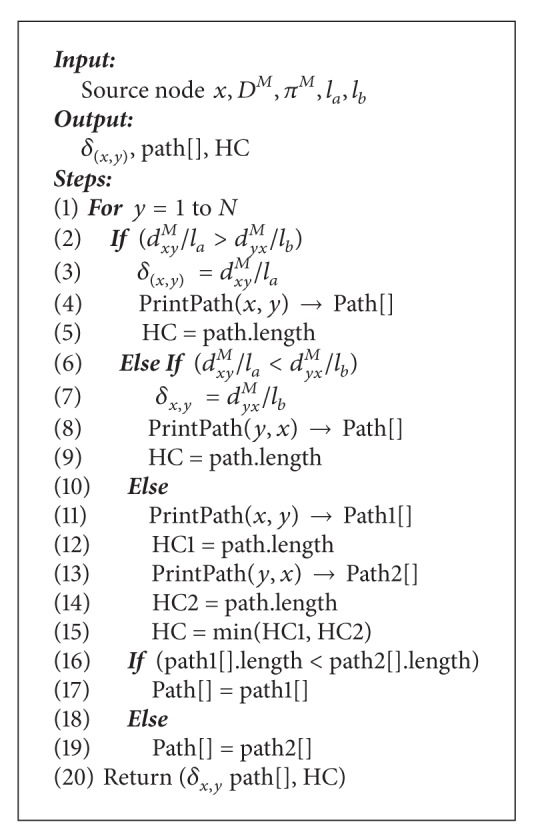
OQRA.

**Algorithm 3:**
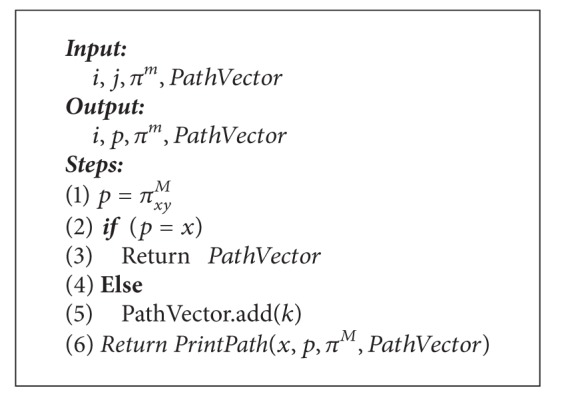
PrintPath.

**Algorithm 4:**
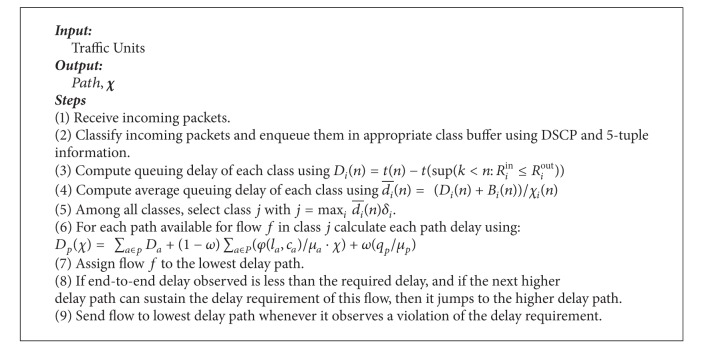
QMPF.

**Table 1:** A comparison between OQRA and PQRA.

Point of view	OQRA	PQRA
Computation	Every time a request initiated.	Precomputes paths from a source to all destinations.

Scalability	Limited.	Large-scale networks.

Suitability	When requests arise infrequently.	When requests arise very frequent.

Pros	Optimal routing during congestion state.	(i) Saves time. (ii) Better scalability. (iii) Improved load balancing.

Cons	Puts excessive time on packet processing.	(i) Path oscillation. (ii) Nonoptimal routing during congestion. (iii) Resources consumption. (iv) Complexity.

**Table 2:** DSCP Assignation for Internet traffic.

Service Group	Service Category	Major Service classes	Flow C/Cs	QoS Metrics					DSCP Value	Traffic class	Minor Service classes
				Timeliness			B.W	Reliability			
				RT (sec)	Delay (ms)	Jitter (ms)			
	Best Effort	Default	Unclassified	U	U	U	U	U	000000	CS0-Default	Everything else
User oriented	Data oriented	Low priority	Symmetric	1 sec	<200	N/A	Elastic	Zero Loss	001000	CS1	Internet Chat
		Interactive	(i) short lived (ii) Low Latency (iii) Asymmetric	2–5	250–400	N/A	Elastic	Zero Loss	010010 010100 010110	AF23 AF22 AF21	Telnet Web Browsing Enhanced Web
		Bulk Transfer	(i) long-lived (ii) High Throughput (iii) Asymmetric	2–5	Low-Medium	N/A	Elastic	Zero Loss	001010 001100 001110	AF11 AF12 AF13	E-Mail FTP Billing transfer
	Media oriented										
	Tolerant Real Time Symmetric	Multimedia Conferencing	(i) interactive (ii) group communication (iii) Rate adaptive	Rate adaptive	<150	<400	8 kbps–1 Mbps	Very low loss	100110 100100 100010	AF43 AF42 AF41	Audio Audio graphics video
		Telephony Service	CBR, fixed small packets, Interactive and fast response	<150	<100	<400	8 kbps–1 Mbps	Very low loss	101110 101100	EF EF	VOIP Videophony
	Intolerant Real Time Symmetric	Broadcasting	(i) Inelastic (ii) CBR and VBR	2–5	<150	50–100	56 K–40 M	low loss	011010 011100	AF31 AF32	Broadcast Video Broadcast Audio
		Streaming	(i) Elastic (ii) Variable packet size	2–5	<150	<100	64 k–60 M	low loss	011000	CS3	Streaming media
		Interactive	(i) Inelastic (ii) VBR	2–5	<150	<100	64 k–60 M	low loss	100000	CS4	VoD

Network oriented	Network Control	Routing and control information	Inelastic-Short messages	N/A	1–10 s	N/A	Elastic	Zero Loss	110000	CS6	Routing information
	Operation and Management signaling	OAM	Inelastic-Short messages	N/A	50–100	N/A	Elastic	Zero Loss	010000	CS2	OAM
	Application Control	Signaling Service Class	Inelastic-Short messages	N/A	50–100	N/A	Elastic	Zero Loss	101000	CS5	VOIP Signaling

**Table 3:** Simulation parameters.

Parameter	Value
Number of nodes	10–250
Routing protocol	OSPFxQoS
Simulation time	300 Sec
Packet size	50–100 byte
Traffic flow	TCP—UDP
Session arrival rate	0.02 ms
Traffic sources	CBR

**Table 4:** Ranges and correlations of link weights.

No correlation	Negative correlation
*w*_1_(*i*, *j*) ~ unifrom[1,100] *w*_2_(*i*, *j*) ~ unifrom[1,200]	*w*_1_(*i*, *j*) ~ unifrom[1,50] *w*_2_(*i*, *j*) ~ unifrom[100,200]
	*w*_1_(*i*, *j*) ~ unifrom[50,100] *w*_2_(*i*, *j*) ~ unifrom[1,100]
